# Carbon Dots-Based Fluorescent Sensor for Glyphosate Detection Via Fe^3+^-Mediated Fluorescence Quenching and Recovery

**DOI:** 10.1021/acsomega.5c09330

**Published:** 2025-12-02

**Authors:** Gabriela Fernandes Barreto, Ricardo Mathias Orlando, Fabiano Vargas Pereira

**Affiliations:** Department of Chemistry − 28114 Universidade Federal de Minas Gerais. Av. Antônio Carlos, 6627 - Pampulha, Belo Horizonte CEP 31270-901, MG, Brazil

## Abstract

In this work, four different carbon dots (CDs) were synthesized by hydrothermal carbonization, each from a distinct low molar mass carbon source (ascorbic, citric, maleic, or succinic acid) in combination with the same nitrogen source (ammonium citrate). The carbon nanoparticles were thoroughly characterized using a range of techniques, including fluorescence spectroscopy, UV–Vis spectroscopy, Raman spectroscopy, transmission electron microscopy (TEM), Fourier-transform infrared spectroscopy (FTIR), and X-ray diffraction (XRD). The optical and structural characterization revealed that the choice of precursors influences the quantum yield of fluorescence (QY) and emission behavior, while yielding nanoparticles of similar sizes (1.7–2.8 nm). The CDs exhibited excitation-dependent emission, which is attributed to distinct energy levels arising from the presence of various surface functional groups, including hydroxyl, carboxyl, amine, and amide groups. Among the synthesized CDs, those prepared from succinic acid and ammonium citrate (SAC) exhibited the highest selectivity and sensitivity toward Fe^3+^ ions, with a detection limit (LOD) of 0.37 μM. This property was exploited to design an “off-on” sensor for the herbicide glyphosate. After a detailed optimization of parameters for glyphosate detection, which included the incubation time of ferric ions and glyphosate as well as the solutions pH, a LOD value of 0.59 μM was obtained, corresponding to concentrations significantly lower than the maximum allowable concentration by international standards. Finally, the developed sensor was applied to real tap water and urban stream samples, and the results showed its potential to be used in rapid, low-cost, and environmentally friendly tests, making it a promising alternative for monitoring the herbicide in aquatic environments.

## Graphical abstract

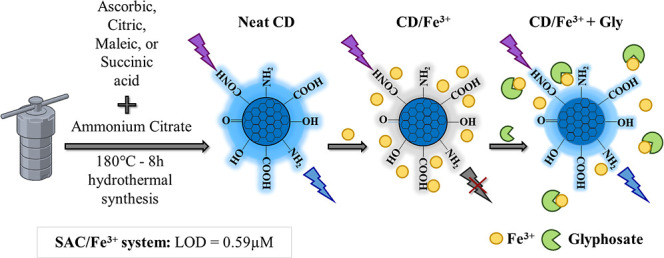

## 1 Introduction

Carbon dots (CDs) represent a relatively new class of carbon-based materials, first reported in 2004,^1^ with a more comprehensive description of their properties presented in 2006.^2^ Typically, they consist of spherical nanoparticles with sizes ranging from 2 to 5 nm, composed of carbon atoms in both sp^2^ and sp^3^ hybridization states.^3,4^ These nanomaterials have been described as structures characterized by a carbonized core surrounded by a shell containing various functional groups, which play a crucial role in determining their chemical reactivity and optical properties.^5,6^ The optical properties of CDs are typically characterized by absorption in the UV or visible region and by a strong and tunable fluorescence emission.^7–9^ A remarkable feature of this fluorescence is its photostability, with the emission wavelength being adjustable mainly through surface chemistry and also by nanoparticle size.^10,11^ Considering these aspects, it is important to emphasize the differences between semiconductor nanocrystals, known as quantum dots (QDs), and CDs. QDs, such as cadmium selenide (CdSe) and cadmium telluride (CdTe), also exhibit dimensions typically ranging from 2 to 10 nm and display properties such as narrow-band fluorescence and high photostability. However, their applications are limited by the presence of heavy metals, which impart toxicity. In contrast, CDs, composed mainly of carbon and oxygen- and/or nitrogen-containing functional groups and free of heavy metals, are notable for their lower toxicity compared with QDs, thereby minimizing environmental risks and sustainability concerns.^12^

In general, it is difficult to establish a definitive rule regarding the toxicity of CDs, as it depends on several factors such as the synthesis route, surface chemistry or doping, dose, and the exposed organism. Nevertheless, many studies have reported that CDs show low or negligible toxicity at low concentrations and in the absence of repeated or prolonged exposure.^13,14^ Furthermore, it is important to highlight that the “eco-friendliness” often attributed to CDs generally refers to the synthetic route (e.g., hydrothermal carbonization), the use of mild reaction conditions, and/or biomass-derived precursors.

All of the features described above, combined with high water solubility, ease of surface chemical modification, low toxicity, relatively low production cost, and straightforward scalability, have attracted considerable research interest in these carbon nanomaterials, both from a fundamental perspective and for diverse practical applications.^15,16^ Among the most promising applications of CDs, we may highlight bioimaging and nanomedicine, where they act as cellular markers and drug carriers;^17,18^ photocatalysis and renewable energy, including solar cells;^19,20^ electronics and optoelectronic materials, comprising LEDs and related devices;^21^ and environmental remediation, where they enable contaminant detection and removal.^22,23^

Considering the preparation methods of CDs, they are generally classified into two main categories: top-down and bottom-up approaches.^3,24^ Top-down strategies consist of breaking larger carbon structures, such as graphite or carbon nanotubes, into nanoscale fragments.^21^ In contrast, bottom-up approaches rely on the growth of carbon nanostructures from low-molar-mass precursors, through a cascade of processes, including polymerization, dehydration, and carbonization, that ultimately lead to the formation of nanoparticles.^5^ Typical bottom-up strategies include hydrothermal, solvothermal, and microwave-assisted reactions.^21^ These latter methods have been increasingly employed in recent years due to their simplicity, relatively low cost, and the possibility of finely tuning certain nanoparticle properties, such as surface chemistry, solubility, optical characteristics, and the functionality of the resulting nanomaterials for specific applications.^25^

Glyphosate is a broad-spectrum organophosphorus herbicide, extensively used worldwide, with the ability to chelate metal ions through its carboxyl and phosphonate groups. While some studies suggest that glyphosate exhibits low toxicity to humans and wildlife, emerging evidence indicates potential adverse effects. These include endocrine disruption in mammals,^26^ developmental toxicity in early life stages of aquatic organisms,^27^ alterations in gut microbiota composition,^28^ and more recently, carcinogenic effects have been suggested,^29^ highlighting the need for accurate, reliable, and sensitive detection methods.

Traditional techniques for glyphosate detection, such as chromatography and mass spectrometry, although accurate, require expensive instrumentation and highly specialized personnel.^30,31^ Therefore, there is a need to develop detection methods for this herbicide that are more practical, rapid, low-cost, and environmentally friendly. In this context, fluorescent CDs have emerged as a practical and powerful solution to this challenge, offering fast and sensitive detection, while minimizing environmental impact and demonstrating potential for on-site or real-time monitoring.

In this work, we present the synthesis and characterization of different CDs, including precursors combinations not previously reported, thereby expanding the chemical diversity available for sensor design. Direct correlations between precursor chemistry and the optical and sensing performance are also presented. In addition, a systematic study was carried out to determine the role of important factors such as ionic strength, pH, and the choice of metal ions in influencing sensing performance. Notably, we employed Fe^3+^ ions in an “off-on” detection system for glyphosate, in contrast to most previous studies that rely on Cu^2+^-based systems. We also present detailed studies on the interaction mechanism between Fe^3+^ and the CDs, including discussions based on HSAB theory, static quenching mechanisms evaluated through assays at different temperatures, and lifetime measurements. In summary, the detailed study presented here provides new insights into the design of CDs with tailored properties for herbicide detection, offering a versatile, efficient, and environmentally friendly strategy that enhances the understanding of CD-metal-glyphosate interactions, thereby contributing to the development of sensitive fluorescence-based sensors.

## 2 Experimental Section

### 2.1 Materials

Quinine sulfate, glyphosate, citric acid and Mg­(NO_3_)_2_·6H_2_O were purchased from Sigma-Aldrich (Saint Louis, USA). Maleic acid, ascorbic acid, Pb­(NO_3_)_2_, Cu­(NO_3_)_2_, and Mn­(NO_3_)_2_ were purchased from Neon (Suzano, Brazil). Succinic acid, ammonium citrate, and CaCl_2_ were obtained from Synth (Diadema, Brazil). Fe­(NO_3_)_3_·9H_2_O was obtained from FMaia (Mogi das Cruzes, Brazil). Ni­(NO_3_)_2_ and CoCl_2_·6H_2_O were purchased from Vetec (São Paulo, Brazil). FeSO_4_ was obtained from Sulfal (Brazil), while HgCl_2_ and ZnSO_4_·7H_2_O were purchased from Merck (Darmstadt, Germany). CrCl_3_ was obtained from Dinâmica (Tatuapé, Brazil). All chemicals were used as received without further purification.

### 2.2 Preparation of the Carbon Dots

Carbon dots were synthesized via the hydrothermal carbonization (HC) method using a Teflon-lined stainless steel autoclave reactor. Initially, 15 mmol of a carbon source (ascorbic, citric, maleic or succinic acid) was mixed with the same amount of the nitrogen source (ammonium citrate) and stirred on a magnetic stirrer until a homogeneous solution was obtained. The solution volume was adjusted to 30 mL, and the reaction was carried out in a 60.0 mL reactor for 8 h at 180 °C. Afterward, the system was allowed to cool naturally, and the dispersion was centrifuged to remove any precipitates, followed by filtration through a 0.22 μm hydrophilic membrane filter. The material was then dried under vacuum in an oven for 72 h. Accordingly, with ammonium citrate used as the nitrogen source for all samples, the CDs prepared with ascorbic, citric, maleic and succinic acid as the carbon source were denoted as AAC, CAC, MAC, and SAC, respectively.

### 2.3 Characterization Methods

The UV–vis absorption spectra were recorded using a Hitachi U-2010 spectrophotometer. Measurements were performed on aqueous CD suspensions in quartz cells with a 1.0 cm optical path length. The photoluminescence spectra were acquired using a Varian Cary Eclipse spectrometer with different excitation wavelengths.

To determine the fluorescence quantum yield (QY) of the samples, UV–vis absorbance spectra were recorded at different concentrations for both quinine sulfate solutions (0.1 M H_2_SO_4_), used as the standard, and CD dispersions. The same CD dispersions and quinine solutions were also analyzed by fluorescence spectroscopy with an excitation wavelength of 340 nm. The QY was then calculated for all samples using the equation

1 $$\phi_{CD}=\phi_{ST}\left(\frac{S_{CD}}{S_{ST}}\right)\left(\frac{\eta_{CD}^{2}}{\eta_{ST}^{2}}\right)$$

In this equation, ϕ represents the quantum yield, CD refers to the carbon dot sample, ST denotes the standard quinine, *S* is the slope of the fluorescence versus absorbance curve, and η is the refractive index.^32^

For transmission electron microscopy (TEM) imaging of the CDs, a dilute dispersion of the samples was deposited onto an ultrathin carbon film. The images were acquired using a Tecnai G2-20 SuperTwin FEI microscope operated at 200 kV. Raman spectral analysis was performed using a Witec alpha300 RA spectrometer. Aqueous suspensions of the samples were deposited onto a glass slide and allowed to dry at room temperature before measurement. X-ray diffraction (XRD) measurements were carried out on a dried CD sample using an Anton Paar XRDynanic-500 diffractometer, operating at 40 kV and 50 mA. Data were collected over a 2θ range of 5–80° with a scanning speed of 0.01° s^–1^. Elemental analysis was performed using a PerkinElmer 2400 Series II CHNS/O Analyzer. Time-resolved fluorescence measurements were carried out using a Horiba–Jobin Yvon FL3-221 spectrofluorometer equipped with a Horiba PPD 850 ps photon detector and a nanoLED excitation source operating at 340 nm. FTIR analysis was performed using a PerkinElmer Spectrum RXI spectrometer in ATR mode, covering a wavenumber range of 400–4000 cm^–1^.

### 2.4 Evaluation of the Obtained Carbon Dots as Sensors for Glyphosate

#### 2.4.1 Effect of Ionic Strength and Metal Ion Detection

First, the fluorescence intensity of the synthesized CDs was evaluated in aqueous KCl solutions with concentrations ranging from 0 to 3.0 mol·L^–1^.

Then, to evaluate the selectivity of the CDs toward different ions, a selectivity experiment was conducted using 12 solutions of individual metal ions: Ca^2+^, Co^2+^, Cr^3+^, Cu^2+^, Fe^2+^, Fe^3+^, Hg^2+^, Mg^2+^, Mn^2+^, Ni^2+^, Pb^2+^, and Zn^2+^, each at a concentration of 0.01 mol·L^–1^. In each assay, 100 μL of each metal ion solution was added to a 2.5 mL dispersion of CDs at a concentration of 20 ppm, and the mixtures were analyzed after a 3 min incubation period. Fluorescence intensity was then measured, and the metal ion that caused the highest fluorescence quenching was selected for sensor development. Subsequently, to assess the selectivity of the chosen metal ion in the presence of competing ions, the analysis was repeated by adding the remaining metal ions to the assay along with the selected ion.

To evaluate the sensitivity of Fe^3+^ ion detection, quantitative studies of the PL intensity response of the CDs to Fe^3+^ were carried out by adding increasing concentrations of the ion in two distinct concentration ranges: from 20 to 500 μM and from 0.08 to 5 μM.

#### 2.4.2 Optimization of Glyphosate Detection Conditions: Incubation Time and pH Studies

The optimal conditions for the CD-based nanosensor were determined by analyzing response time and pH effects. The quenching by Fe^3+^ and fluorescence recovery with glyphosate were studied separately.

For the time response study, and considering the quenching step, 30 μL of Fe^3+^ solution (0.01 mol·L^–1^) was added to 2.5 mL of CD dispersion (20 mg·L^–1^), and the fluorescence intensity was measured at various time intervals. For the recovery step, 30 μL of glyphosate solution (0.01 mol·L^–1^) was added to the prequenched CD/Fe^3+^ system, and fluorescence was recorded at different time intervals to monitor signal restoration.

To evaluate the effect of pH, CD dispersions (2.5 mL, 20 mg·L^–1^) were prepared and adjusted to different pH values by adding appropriate volumes of HCl or NaOH (0.1 mol·L^–1^). Subsequently, 100 μL of Fe^3+^ and 100 μL of glyphosate solutions (0.01 mol·L^–1^) were added to the dispersions at different pH values. The influence of pH on fluorescence quenching (Fe^3+^ addition) and fluorescence recovery (glyphosate addition) was assessed by measuring the PL intensity under these conditions.

#### 2.4.3 Study of Glyphosate Detection under Optimized Conditions

After determining the optimal conditions for glyphosate detection, the sensor was prepared by mixing 2.5 mL of a carbon dot dispersion at 20 ppm (adjusted to the ideal pH for each sample), with 50 μL of an Fe^3+^ solution (0.01 mol·L^–1^). The mixture was then incubated for the previously determined optimal time. For the herbicide analysis, different volumes of a glyphosate solution (0.01 mol·L^–1^) were added to the CD/Fe^3+^ dispersion. The mixture was incubated for the established optimum time, and the fluorescence intensity was subsequently measured.

#### 2.4.4 Evaluation of the Sensor in Real Water Samples

To assess the applicability of the sensor in real water samples, a 3.0 mL dispersion of a CD sample at 20 ppm was prepared and adjusted to the optimal pH previously determined. Then, 30 μL of a 0.01 mol·L^–1^ Fe^3+^ solution was added, and the mixture was stirred for 3 min to ensure proper homogenization and interaction between the components. Real water samples, including tap water and a local urban stream, were filtered through a 0.22 μm membrane, spiked with different volumes of a 0.01 mol·L^–1^ glyphosate solution, and incubated for 10 min prior to fluorescence measurements.

## 3 Results and Discussion

### 3.1 Characterization of the Different Carbon Dots

Different CDs were synthesized to investigate the influence of distinct precursors and surface functional groups on their properties, with a particular focus on their optical behavior. Subsequently, the photoluminescence behavior of the CDs was evaluated under different ionic strengths as well as their ability to selectively interact with small amounts of specific metal ions. The selective and sensitive interaction with a particular metal ion was then employed to develop a nanosensor for glyphosate detection.

Figure 1a–f summarize the optical properties of the obtained CDs, including photographs illustrating their appearance under visible and UV light, as well as their UV–vis absorption and fluorescence spectra. Figure 1a shows the CDs under visible light, while Figure 1b displays their fluorescence under UV illumination (λ = 365 nm). Under visible light, the samples exhibit a transparent appearance with a yellowish-brown or greenish coloration, while under UV light they emit blue fluorescence. Figure 1c–f show the absorption spectra and the fluorescence emission profiles of the different carbon nanoparticles, recorded at various excitation wavelengths (λ_exc_ = 300–400 nm). The UV–vis absorption spectrum of the AAC sample exhibited a band around 280 nm (Figure 1c), attributed to the characteristic π → π* transition of conjugated C=C bonds in aromatic π-systems (sp^2^ domains).^33,34^ In contrast, the absorption spectra of the CAC, MAC, and SAC samples exhibited only bands with maxima around 320 nm, corresponding to the characteristic *n* → π* transition of oxygen- and nitrogen-containing groups, such as those present in carboxylic acids and amides.^22,30^ At the same time, a subtle shoulder in the UV–vis spectrum of the AAC sample also indicates the presence of *n* → π* transitions in this sample. Among the carbon sources used in the synthesis of the CDs, only ascorbic acid contains a cyclic structure with a lactone ring. Although this ring is not aromatic, its presence, along with multiple hydroxyl groups, may promote dehydration and cyclization reactions during the carbonization process, favoring the formation of π-conjugated domains. Such conjugated structures are essential for the π → π* electronic transition, which is responsible for the absorption band observed at 280 nm for the sample AAC.

**Figure 1:**
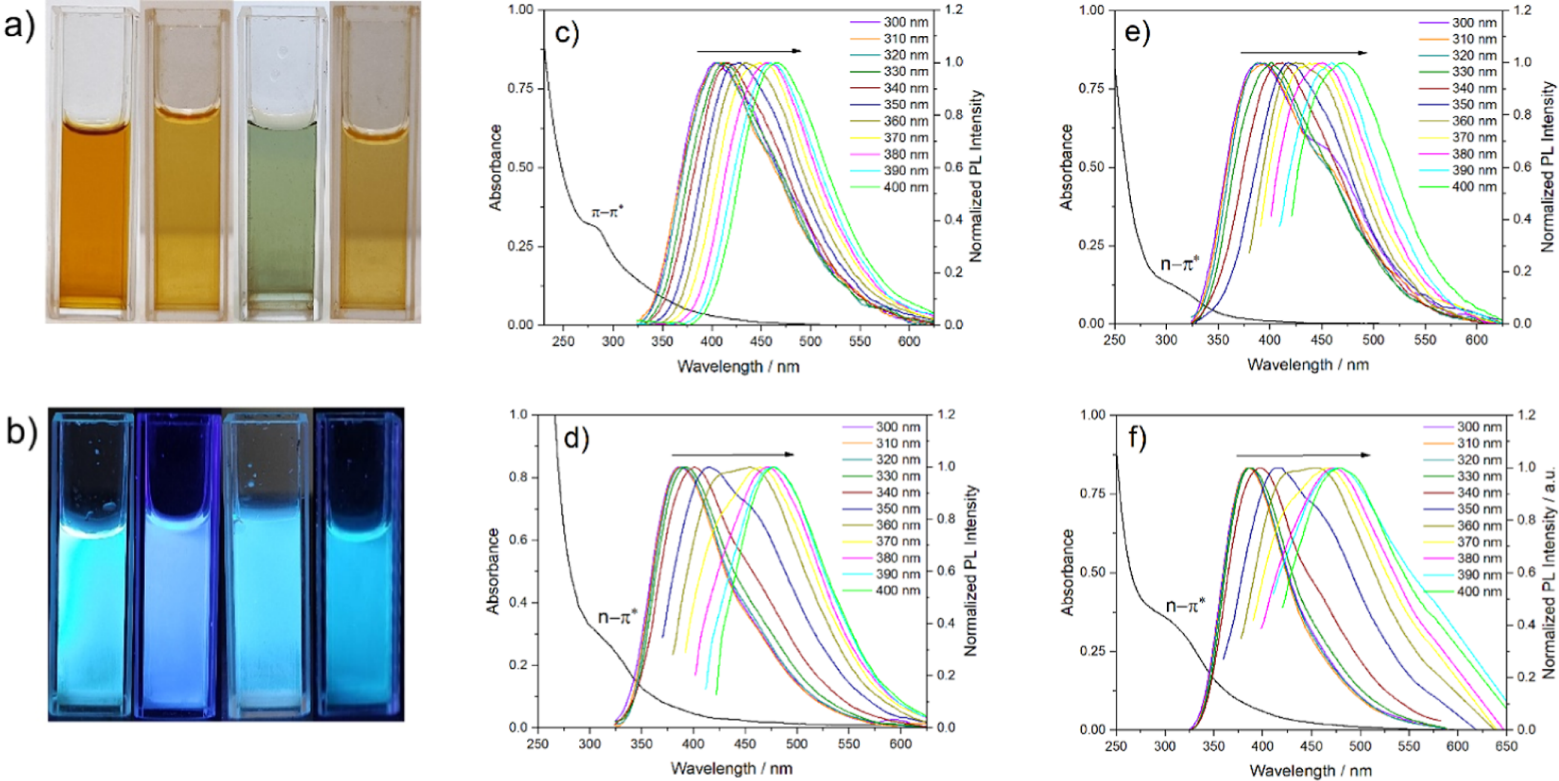
Optical characterization of the synthesized CDs. (a) Photographs of CD solutions under visible light and (b) under UV illumination (λ = 365 nm), with the samples from left to right in the following order: AAC, CAC, MAC, and SAC. (c–f) UV–vis absorption spectra (left) and excitation-dependent fluorescence emission spectra (right) of AAC, CAC, MAC, and SAC samples, respectively. Emission spectra were recorded at excitation wavelengths ranging from 300 to 400 nm, in 10 nm intervals.

Figure 1c–f also show the normalized PL spectra of the CDs, obtained by varying the excitation wavelength in 10 nm intervals within the 300–400 nm range. The fluorescence spectra exhibit excitation-dependent emission behavior, with the emission peak gradually shifting toward the red region as the excitation wavelength increases, as indicated by the arrows in the spectra. This phenomenon can be attributed to surface states arising from the presence of different functional groups on the surface of the CDs, which are associated with distinct energy levels.^35,36^ The same fluorescence spectra are shown in Figure S1 without normalization. The maximum fluorescence emission intensities were observed for the CAC and SAC samples upon excitation at 320 nm, and for the AAC and MAC samples at 340 and 330 nm, respectively. These excitation wavelengths, corresponding to maximum emissions at 416, 391, 401, and 386 nm for the AAC, CAC, MAC, and SAC samples, respectively, were subsequently used to evaluate their sensing performance toward glyphosate, as discussed in a later section.

The fluorescence quantum yield (QY) is an important characteristic of CDs, as a sufficiently high QY value is typically desirable for several applications of these nanoparticles, such as in fluorescent markers or sensors.^37^ Thus, the QY values were determined from the linear relationship between integrated PL intensity and absorbance at different sample concentrations, by comparing the slopes of these curves with that of the quinine sulfate standard (Figure S2). The QY values obtained for the samples are presented in Table 1, along with the corresponding carbon and nitrogen sources.

**Table 1:** Precursor Combinations for CD Synthesis, Sample Names, and the Corresponding Fluorescence Quantum Yield (QY)

C source	N source	sample	QY/ %
ascorbic acid	ammonium citrate	AAC	9.3
citric acid		CAC	21.6
maleic acid		MAC	12.0
succinic acid		SAC	21.6

The correlation between QY and the nature of the precursors remains a subject of debate in the literature, as the fluorescence properties of CDs often result from complex reaction pathways.^5,38^ Nevertheless, it is generally accepted that the initial reactions play a decisive role in determining the final structure and optical properties of the CDs.^34,35^ All samples in this study share the same nitrogen source (ammonium citrate); therefore, the observed differences can be attributed to the choice of carbon precursor. The CAC and SAC samples, obtained from citric acid and succinic acid, respectively, are aliphatic poly­(carboxylic acid)­s that promote surfaces rich in carboxyl groups (–COOH). In combination with the nitrogen source, this facilitates the formation of C–N bonds and surface amide groups, enhancing the passivation of surface traps and reducing nonradiative pathways, which accounts for the relatively high QY of these two samples. In addition, carboxylic acids are well-known to promote high QY values.^39,40^ Citric acid, containing three carboxyl and one hydroxyl group, facilitates the formation of conjugated carbon domains while simultaneously providing surface passivation, thereby minimizing nonradiative recombination and enhancing photoluminescence.^15^ This is consistent with the high QY observed for the CAC sample (21.6%). A QY of 21.6% was also obtained for the SAC sample, synthesized from succinic acid and ammonium citrate. The favorable molecular structure of succinic acid, with two accessible carboxylic groups and low steric hindrance, likely facilitates early stage reactions such as amidation with ammonium citrate, improving nitrogen incorporation and further contributing to the increased QY.^34,35^ In the MAC sample (maleic acid), the presence of the double bond alters the reaction pathways, potentially reducing the efficiency of *N*-passivation and leading to a less uniform distribution of surface functional groups (C–N, COOH), thereby leaving more sites susceptible to nonradiative recombination.^41^ In addition, the unsaturation promotes side reactions that increase heterogeneity and generate electronic defects, which act as nonradiative sinks and can account for the intermediate QY. In the AAC sample (ascorbic acid), although some nitrogen incorporation is possible, ascorbic acid is chemically unstable and reducing. During synthesis, the oxidation and dehydration of ascorbic acid generate a complex mixture of fragments and surface groups that may hinder uniform and efficient nitrogen passivation, resulting in a higher density of nonradiative traps. Furthermore, its tendency to produce highly oxidized products and less-ordered structures leads to small or fragmented sp^2^ domains, or a highly amorphous matrix, conditions that favor nonradiative recombination and, consequently, a lower QY.^42^

Figure 2a–d present transmission electron microscopy (TEM) images of the different CDs obtained. The micrographs reveal well-dispersed nanoparticles with predominantly spherical morphology. Due to the relatively low concentration of CDs in some images and their low contrast with the carbon grid, red circles were added to facilitate their visualization. The average particle size and size distribution were determined through image analysis using ImageJ software, based on measurements of 100 particles per sample. The mean diameters were found to be 2.2 nm for AAC, 1.7 nm for CAC, 2.8 nm for MAC, and 2.7 nm for SAC, as shown in the size distribution histograms presented in Figure S3. The size distributions are relatively narrow, and the particle sizes are overall similar across samples, indicating that the synthetic approach yields consistent nanostructures, independent of the precursor employed.

**Figure 2:**
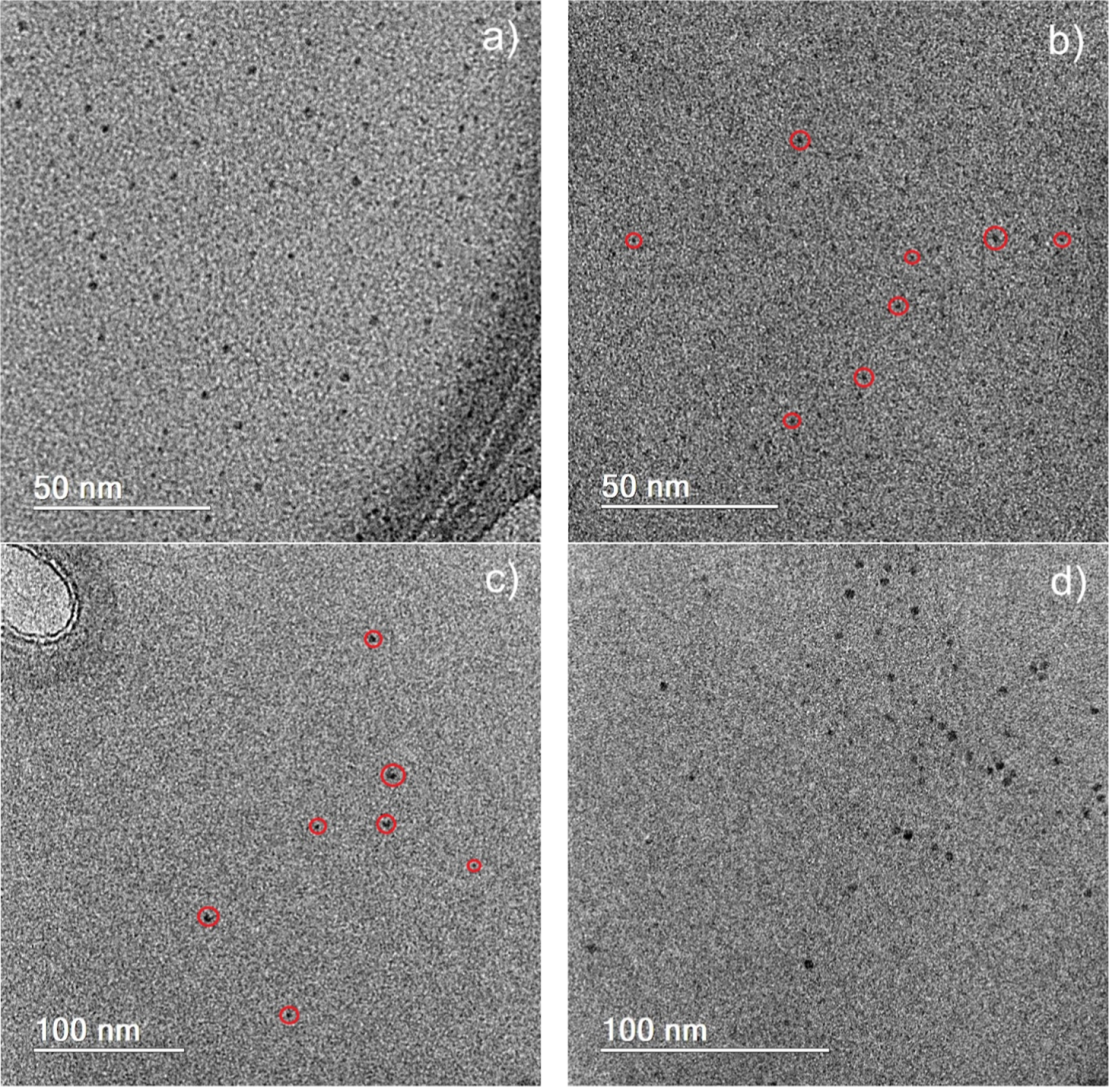
TEM images of the carbon dots obtained from different precursors: (a) AAC, (b) CAC, (c) MAC, and (d) SAC.

Figure S4 presents the Raman spectra for all the CD samples, which exhibit two characteristic peaks at approximately 1360 and 1600 cm^–1^. The peak around 1360 cm^–1^, known as the *D* band, is primarily associated with disordered carbon structures, reflecting the presence of structural defects and surface functional groups.^43^ In contrast, the peak near 1600 cm^–1^, known as the *G* band, corresponds to the *E*_2_g vibrational mode of sp^2^-hybridized graphitic carbon, representing more ordered graphitic domains within the CDs.^38^ The intensity ratio between the *D* and *G* bands (ID/IG) is commonly used to estimate the degree of graphitization or structural order in carbon-based nanomaterials.^44^ Since the *G* band corresponds to ordered graphitic domains, lower ID/IG ratios indicate a higher degree of graphitization. The ID/IG intensity ratios for AAC, CAC, MAC, and SAC were 0.63, 0.78, 0.68, and 0.79, respectively, indicating a relatively high degree of graphitization in all samples.

X-ray diffraction (XRD) analysis of the CD samples (Figure S5) revealed a single broad peak centered at approximately 2θ = 20.0°, commonly associated with disordered carbon structures and an expanded interlayer spacing compared to the 2θ = 26.0° typically observed for the (002) plane in crystalline graphite.^45^ The broad nature and shifted position of this peak suggest that the CDs are composed of small sp^2^-hybridized graphitic domains embedded within largely disordered carbon matrices. Because XRD is sensitive only to long-range crystalline order, it alone cannot provide a definitive assessment of whether the structures are amorphous or semicrystalline. However, when considered together with TEM images, in which no graphitic domains were clearly observed, these results indicate that the CDs possess a predominantly amorphous character.

The results of the elemental analysis, presented in Table S1, indicate that all samples contain more than 50% carbon and approximately 10% nitrogen, which is consistent with the expected composition of *N*-doped carbon dots. The infrared spectra (Figure 3) of the CD samples exhibit several characteristic bands, some of which are common to all samples. All spectra show a broad band between 3000 and 3400 cm^–1^ range, attributed to O–H and N–H stretching vibrations, along with bands between 2927 and 2937 cm^–1^, corresponding to C–H stretching vibrations of alkyl (sp^3^-hybridized carbon) groups.^46^ Bands assigned to C=O stretching vibrations of carboxylic acids and amides are also observed in all samples, with slight variations in position: 1702–1735 cm^–1^ for carboxylic acids and 1650–1682 cm^–1^ for amides.^47^ Additionally, the band between 1400 and 1407 cm^–1^, observed in all spectra, can be attributed to the symmetric stretching of C–O bonds from carboxylate groups on the CD surface.^41^

**Figure 3:**
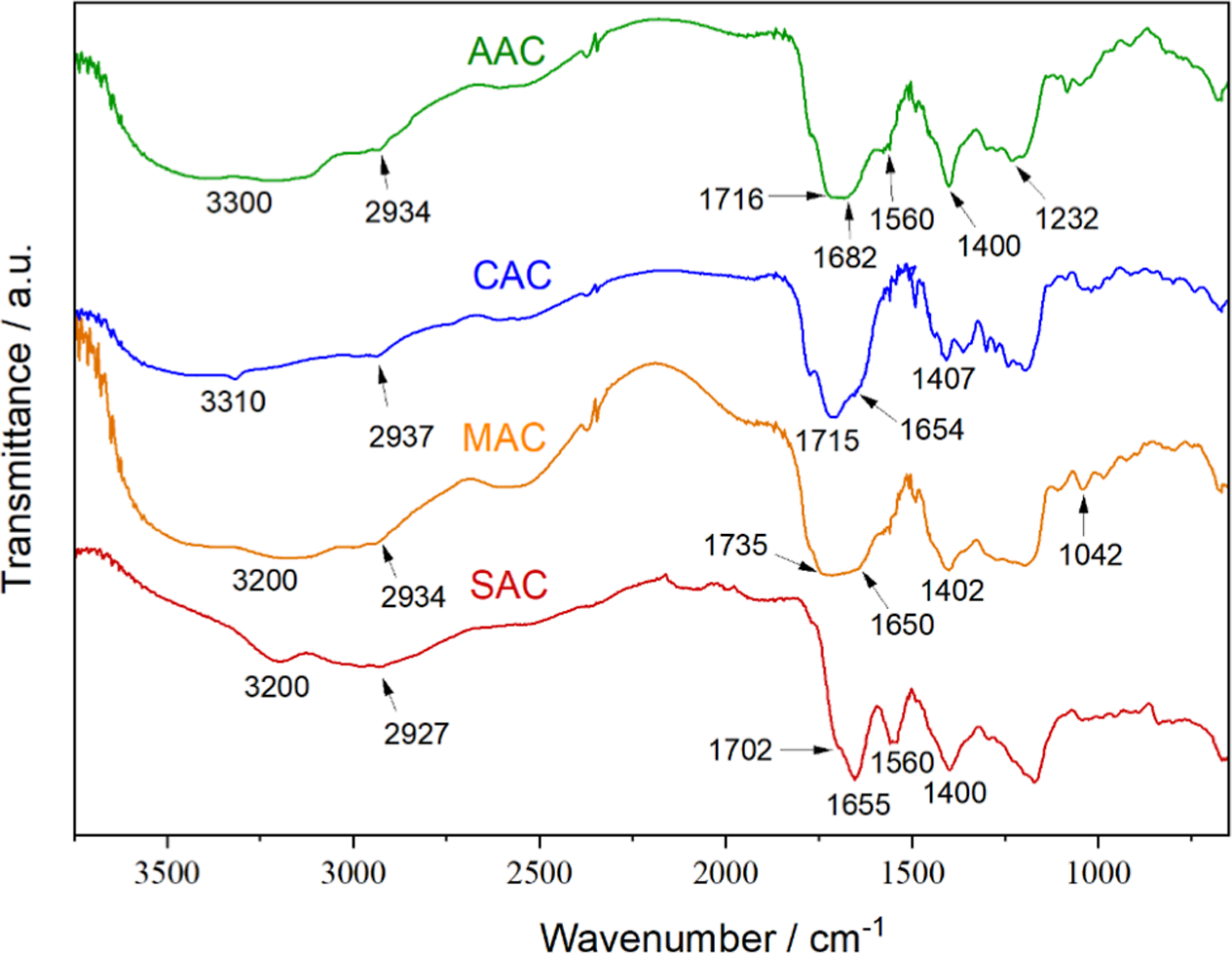
FTIR spectra of the CDs.

Other bands appear more specifically in individual samples. For instance, both AAC and SAC exhibit a band at 1560 cm^–1^, which may be attributed to aromatic C=C vibrations.^41^ The band at 1232 cm^–1^ in the AAC spectrum is likely associated with C–O stretching and/or C–N bonding. The MAC sample, in turn, displays a band at 1042 cm^–1^, corresponding to the C–O stretching vibration of alcohols.^48^

### 3.2 Evaluation of the Synthesized CDs as Nanosensors for Glyphosate

The search for alternative and low-cost methods for glyphosate detection has been reported in the literature and includes the use of nanoparticles such as gold nanoparticles (AuNPs) and CDs. In AuNP-based approaches, stabilized nanoparticles are typically employed, which aggregate upon interaction with the herbicide, inducing a color change detectable by UV–vis spectrophotometry or by the naked eye.^49,50^ In this context, CDs can provide an alternative for glyphosate detection without the use of heavy metals and with lower synthesis costs. Among the various sensing strategies employing these carbon nanoparticles, “off-on” systems are particularly attractive due to the intrinsic fluorescence of the carbon nanoparticles, which allows fluorescence quenching (“off”) or recovery (“on”) upon interaction with the target analyte. This section presents the development and application of a CD-based sensor, combined with a metal ion, for glyphosate detection in water. To determine the optimal detection conditions, the main factors affecting the optical properties of the CDs were systematically evaluated, including ionic strength, selectivity and sensitivity to metal ions, pH, and incubation time.

#### 3.2.1 Effect of Ionic Strength and Metal Ion Detection Studies

The ionic strength of the medium can directly affect the optical properties of CDs, and consequently, the stability of the nanoparticle dispersion is a critical factor in assessing their potential for practical applications.^51^ In this study, the fluorescence intensity of the synthesized CDs was evaluated in KCl solutions with concentrations ranging from 0 to 3.0 mol·L^–1^. The normalized fluorescence intensity of the samples as a function of KCl concentration is presented in Figure S6. The results showed minimal variation in fluorescence intensities with respect to salt concentration for the samples AAC and SAC, indicating good stability and resistance to ionic variations. For the CAC and MAC samples, a greater variation in fluorescence was observed, particularly at concentrations above 1.0 mol·L^–1^, with a more pronounced effect in the MAC sample.

A detailed analysis of the selectivity and sensitivity of the synthesized CDs toward various metal ions enables the identification of the most responsive CD/metal ion pairs, characterized by pronounced and distinct fluorescence changes. This is essential for developing “off-on” type sensors and for the selection of suitable systems for subsequent target analyte detection. To evaluate the selectivity of the CDs, 12 different metal ions were studied: Ca^2+^, Co^2+^, Cr^3+^, Cu^2+^, Fe^2+^, Fe^3+^, Hg^2+^, Mg^2+^, Mn^2+^, Ni^2+^, Pb^2+^, and Zn^2+^, each at a concentration of 0.01 mol·L^–1^. A volume of 100 μL of the metal ion solution was added to 2.5 mL of the CD dispersion (20 ppm). The quenching efficiency was calculated using the following equation.^52^

2 $$QE=\frac{F_{0}-F}{F_{0}}\times 100$$

In this equation, QE represents the quenching efficiency, *F*_0_ is the fluorescence intensity without metal ions, and *F* is the fluorescence intensity measured in the presence of metal ions. The results are presented in Figure 4. In general, the CDs exhibited selectivity toward Fe^3+^ ions, although with different quenching efficiency (QE) values. The AAC and MAC samples, while exhibiting considerable selectivity for Fe^3+^, showed the lowest quenching efficiency, with fluorescence intensity reductions of approximately 40% (AAC) and 50% (MAC), as calculated using eq 2. In contrast, the CAC and SAC samples exhibited more pronounced quenching effects in the presence of Fe^3+^, with QE values of 81 and 79%, respectively. However, it is noteworthy that the CAC sample also showed slight fluorescence decreases in the presence of other ions, such as Co^2+^, Cu^2+^, Fe^2+^, Hg^2+^, and Pb^2+^, whereas the SAC sample demonstrated greater selectivity for Fe^3+^, showing negligible changes with the other metal ions. Thus, the SAC sample was identified as the most selective toward Fe^3+^ ions, combining high quenching efficiency (QE = 79%) with superior selectivity for ferric ions.

**Figure 4:**
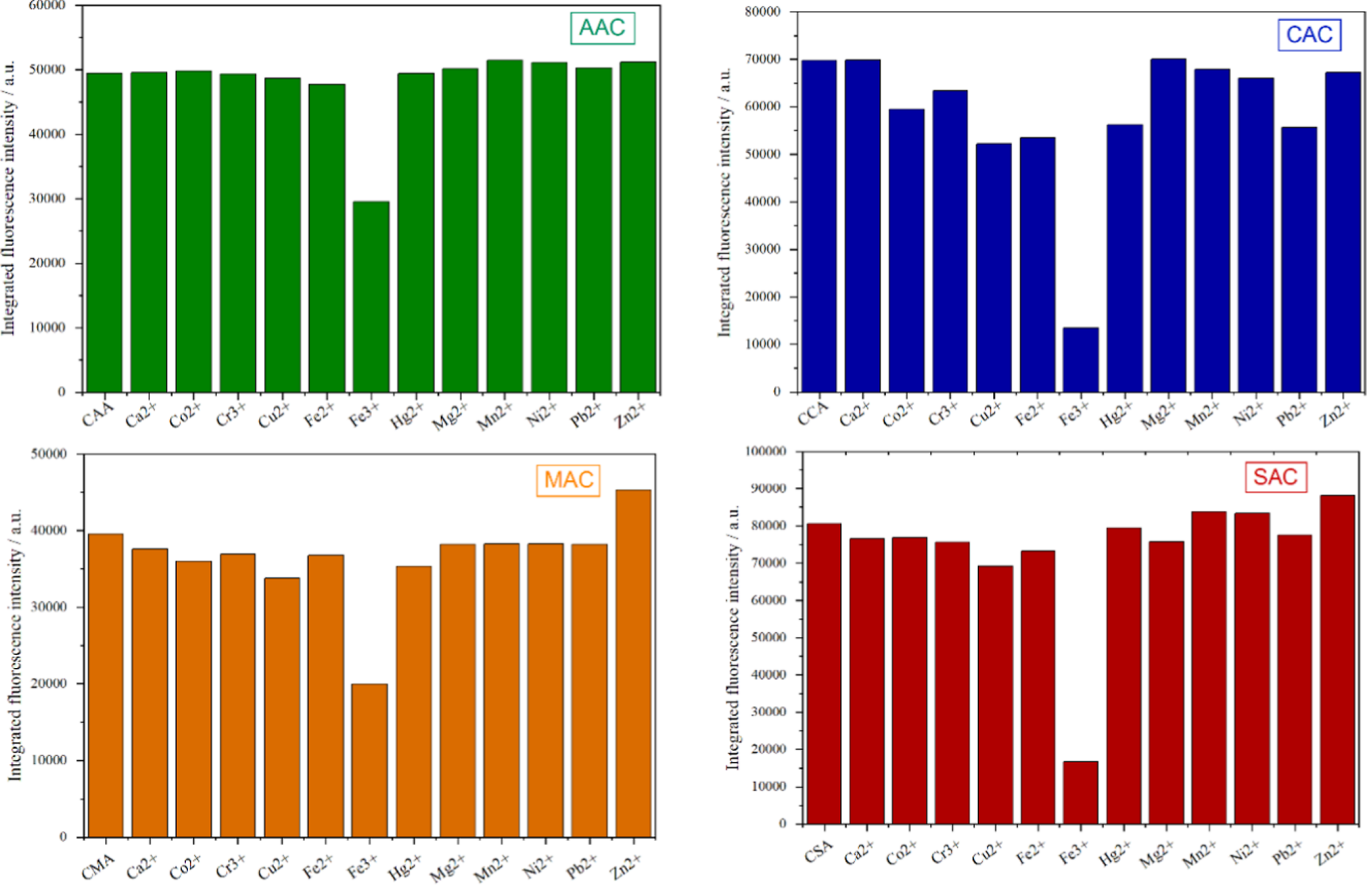
Relative PL intensity of neat CDs compared to PL intensities of CD samples after addition of various metal ions.

In addition, to evaluate possible interferences in the quenching response, the control system (CD/Fe^3+^) was compared with that containing both Fe^3+^ and an additional metal ion (CD/Fe^3+^ + ion). As shown in Figure S7, the addition of other ions did not significantly affect the fluorescence intensity, indicating the absence of competitive interference in the quenching process. These findings confirm that the system maintains its selectivity even in the presence of other potential interfering metal ions. Overall, the results demonstrated that the CDs exhibit high selectivity toward Fe^3+^ ions compared to other ions. Consequently, Fe^3+^ was selected as the target ion for sensor development.

Quantitative fluorescence response studies of the CDs toward ferric ions were conducted over two concentration ranges: a higher range (200–500 μM) and a lower range (0.08–5 μM). For the higher concentration range, Figure S8 shows the fluorescence emission profiles of the CD samples, excited at the wavelength corresponding to their maximum emission intensities: 320 nm for the CAC and SAC samples, and 340 and 330 nm for the AAC and MAC samples, respectively. As the Fe^3+^ concentration increased, the fluorescence intensity progressively decreased, indicating a quenching process directly correlated with the amount of ferric ions present in the solution. However, to employ the fluorescent nanocarbons as selective and sensitive sensors for glyphosate, it is essential that their fluorescence exhibits detectable changes even in the presence of small amounts of Fe^3+^ ions. Such sensitivity allows the detection of low concentrations of the analyte (glyphosate) in the second stage of the “off-on” detection system, as glyphosate is expected to bind the same ferric ions that interact with the CDs in the first stage. Thus, the same study was carried out within the lower concentration range of Fe^3+^ ions (0.08–5 μM). Figure 5 shows a linear relationship between the fluorescence intensity ratio (*F*/*F*_0_) and the concentration of Fe^3+^ ions for the samples, where *F* and *F*_0_ represent the fluorescence intensities in the presence and absence of Fe^3+^, respectively. This behavior reflects a gradual decrease in the PL intensity at the maximum emission wavelength as the Fe^3+^ concentration increases. Among the samples, SAC exhibited the strongest linear relationship, with a correlation coefficient (*R* ^2^) of 0.9993 within this concentration range. Based on these curves, the limit of detection (LOD) for Fe^3+^ was calculated using the 3δ/*S* criterion, where δ represents the standard deviation, and *S* is the slope of the curve. The LOD values obtained for all samples were 1.07 μM, 0.60 μM, 0.55 μM, and 0.37 μM for AAC, CAC, MAC, and SAC samples, respectively.

**Figure 5:**
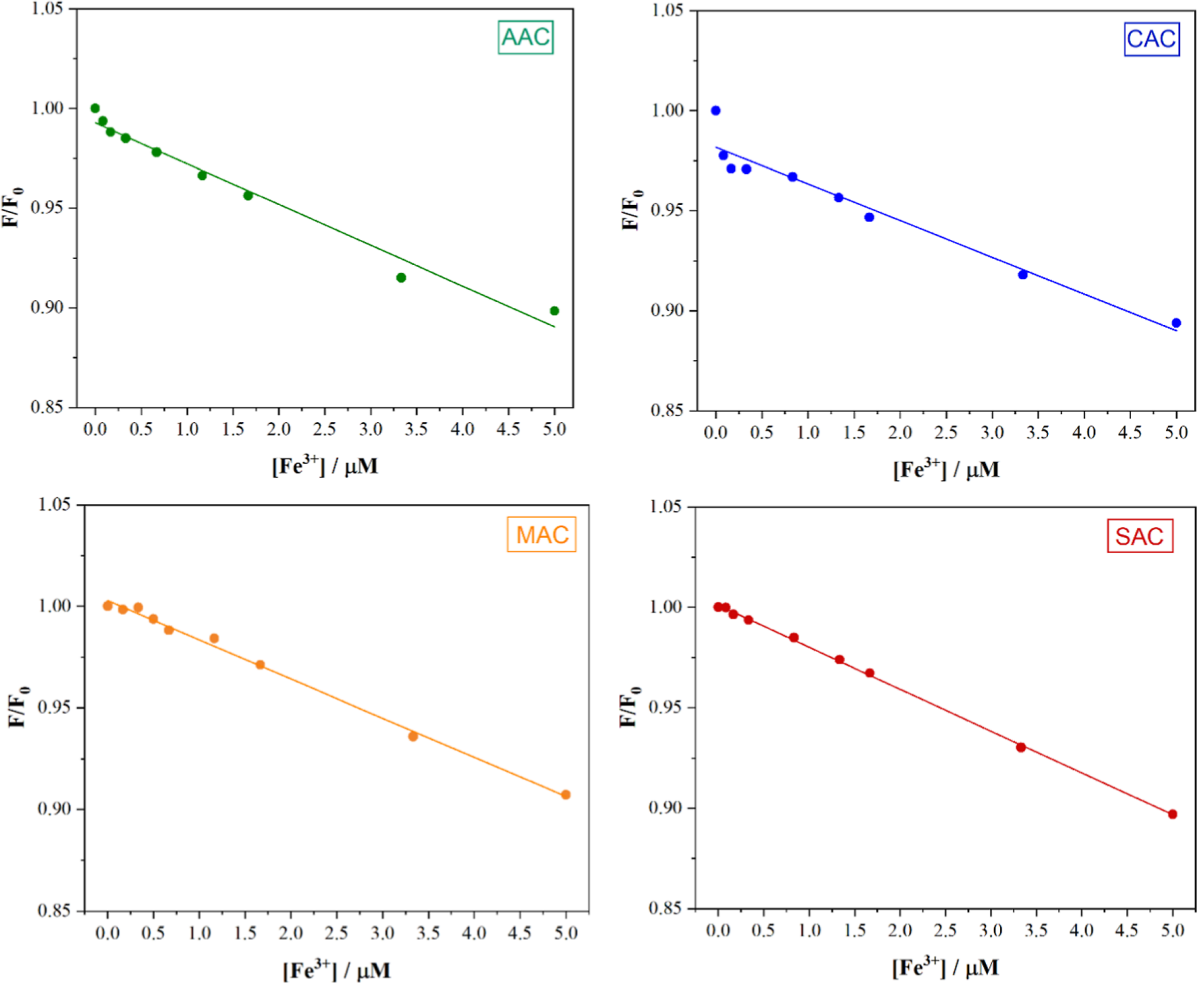
Fluorescence intensity ratio (*F*/*F*_0_) as a function of Fe^3+^ ion concentration in the lower concentration range for the CD samples.

At this point, it is interesting to discuss the preferential interaction of CDs with Fe^3+^ ions and the quenching mechanism between ferric ions and the CDs. According to the literature, Fe^3+^ can strongly interact with and influence the oxygen-containing functional groups in carbonaceous materials.^53^ In our case, the strong interaction between Fe^3+^ and the CD surface can be rationalized based on the Hard and Soft Acids and Bases (HSAB) theory.^54,55^ The ion Fe^3+^, a hard acid, tends to coordinate preferentially with hard bases such as the oxygen atoms of surface carboxylate groups and, to a lesser extent, with the nitrogen atoms of amide/amine groups, which are borderline bases.^55^ This interaction leads to the formation of stable surface complexes with predominantly ionic character, significantly affecting the photoluminescence behavior of the CDs. In contrast, the other metal ions, being less hard or more polarizable, exhibit weaker interactions with the oxygen- and nitrogen-containing groups. Mg^2+^ and Ca^2+^ ions, although also classified as hard acids, have lower charge densities and therefore interact more weakly with the CD surface. The Cr^3+^ ion, in turn, is also a hard acid but tends to coordinate to a lesser extent under the same experimental conditions due to its stronger hydration in solution. These considerations explain the higher quenching efficiency and stronger binding observed experimentally for Fe^3+^ compared with the other cations. This selectivity was exploited for the development of the sensor.

The mechanism of the interaction between the carbon nanoparticle and ferric ions was investigated using the SAC sample, which exhibited the highest selectivity and sensitivity toward Fe^3+^ ions, the strongest linear correlation in Figure 5, and the smallest variation in PL intensity under changes in ionic strength. Fluorescence quenching can occur via two distinct mechanisms: dynamic and static quenching. Dynamic quenching occurs when the excited-state fluorophore interacts with quencher molecules through diffusive collisions during its lifetime. In contrast, static quenching involves the formation of a nonfluorescent ground-state complex between the fluorophore and the quencher^56,57^ To elucidate whether the quenching mechanism in the presence of ferric ions occurs via a static or dynamic pathway, a study was conducted using varying concentrations of Fe^3+^ ions at different temperatures. As shown in Figure S9, the Stern–Volmer plot for the SAC sample exhibits a linear relationship described by the equation

3 $$\frac{F_{o}}{F}=1+K_{SV}.\left[Q\right]$$

In this equation, *F*_0_ */F* represents the ratio between the fluorescence intensities in the absence (*F*_0_) and presence (*F*) of Fe^3+^; *K*_SV_ is the slope corresponding to the Stern–Volmer constant, and [*Q*] denotes the concentration of Fe^3+^ ions.

When quenching follows a dynamic pathway, higher temperatures typically intensify the process, as increased molecular motion promotes more frequent and energetic encounters between the quencher and the fluorophore. As a result, the Stern–Volmer constant (*K*_SV_) tends to rise with temperature.^46^ In contrast, for static quenching, elevated temperatures generally weaken the association between the quencher and the carbon dots, as thermal motion disrupts the ground-state complex. This reduction in complex stability allows partial restoration of photoluminescence, leading to lower *K*_SV_ values at higher temperatures. The Stern–Volmer constants (*K*_SV_) calculated for the SAC sample from Figure S9 were 1.436 × 10^4^, 1.415 × 10^4^, and 1.325 × 10^4^ L·mol^–1^ at 20, 30, and 40 °C, respectively. This gradual decrease in *K*_SV_ with increasing temperature indicates a static quenching mechanism, in which Fe^3+^ ions form complexes on the surface of the CDs.^46^ This interaction is promoted by the coordination of the ions with surface functional groups, such as carboxyl, which act as electron donors to the half-filled 3d orbital of Fe^3+^, resulting in quenching of the system.^58,59^

In addition to the study carried out by varying the Fe^3+^ concentration at different temperatures, time-resolved fluorescence decay measurements were performed for the SAC sample, with the main purpose of investigating the interaction mechanism between the SAC sample and ferric ions. As illustrated in Figure S10, the fluorescence decay curves of the SAC sample and the SAC/Fe^3+^ system exhibit very similar decay profiles, showing no obvious changes among the different samples. The same behavior is observed for the SAC/Fe^3+^/glyphosate system. By fitting the data, the average fluorescence lifetime of the SAC sample was calculated to be 6.23 ns, while that of the SAC/Fe^3+^ system exhibited a slightly shorter value of 5.51 ns. The subsequent addition of glyphosate to the SAC/Fe^3+^ system also caused no appreciable variation, resulting in an average lifetime of 5.87 ns. The slight variations observed in the fluorescence lifetimes, combined with the results from the Stern–Volmer analysis as a function of temperature, indicate that the fluorescence quenching process occurs predominantly through a static mechanism.

#### 3.2.2 Optimization of Glyphosate Detection Conditions

Effective sensor design requires a thorough investigation of the most suitable operating conditions. To determine the optimal parameters for the CD-based nanosensor, both the response time and the ideal pH of the medium were evaluated. The optimization of the operating conditions of the sensor must consider both stages of the “off-on” mechanism. These stages are schematically illustrated in Figure 6, showing first the interaction between the CDs functional groups and ferric ions, responsible for quenching the system, followed by the interaction between Fe^3+^ ions and glyphosate.

**Figure 6:**
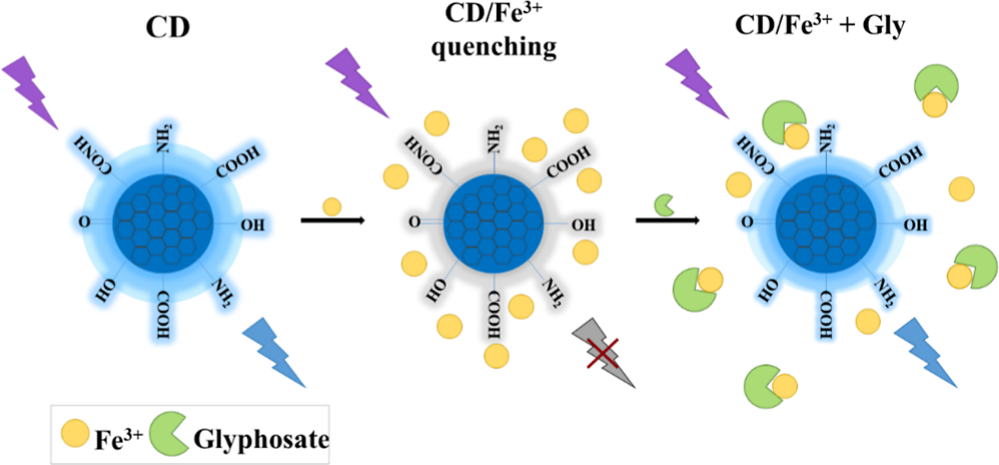
Schematic representation of the glyphosate detection mechanism using the CD/Fe^3+^ system.

Some studies have reported a strong interaction between Fe^3+^ ions and glyphosate.^60,61^ The carboxyl (–COOH) and phosphonate (–PO­(OH)_2_) functional groups of glyphosate can form complexes with Fe^3+^ ions, effectively removing them from the CD/Fe^3+^ system.^50^ This interaction reduces the quenching effect exerted by Fe^3+^ on the fluorescence of the CDs, thereby partially restoring their emission, as illustrated in Figure 6.

For the optimization of the nanosensor, the response time was first assessed in two stages: initially by monitoring the fluorescence quenching induced by Fe^3+^ ions, followed by the assessment of fluorescence signal recovery after the addition of glyphosate. In the quenching step, varying amounts of Fe^3+^ solution were added to the CD dispersion at different time intervals. To recover the PL intensity, predetermined amounts of glyphosate solution (as described in the methodology) were added to the prequenched CD/Fe^3+^ system, and the fluorescence was monitored over time to assess signal restoration. As shown in Figure 7a, a significant decrease in fluorescence is observed within the first minute after the addition of Fe^3+^. *F* and *F*_0_ in Figure 7a represent the fluorescence intensities of the CDs in the presence and absence of ferric ions, respectively. The quenching effect reached a plateau within 3 min for the samples, after which only minor variations in PL intensity were observed. Thus, 3 min was established as the optimal incubation time for the CD/Fe^3+^ system.

**Figure 7:**
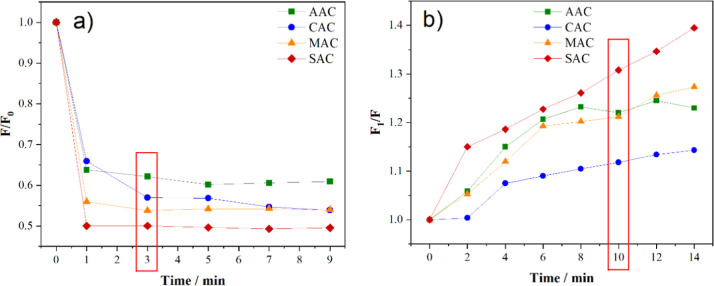
Study of incubation times. (a) Fluorescence quenching of the CD/Fe^3+^ system and (b) fluorescence recovery upon glyphosate addition. The figures highlight the incubation times determined for each step.

As shown in Figure 7b, the addition of glyphosate led to a gradual increase in fluorescence intensity across all evaluated systems, as evidenced by the increase in *F*_1_/*F* values, where *F*_1_ and *F* correspond to the fluorescence intensity of the CDs/Fe^3+^ system in the presence and absence of glyphosate, respectively. Among the samples, SAC exhibited the most intense and continuous fluorescence recovery throughout the entire analysis period, while the other samples showed the majority of their fluorescence restoration within the first 10 min. Thus, to maintain consistent timing across all samples and to maximize the sensor efficiency in terms of analysis time, an incubation period of 10 min with glyphosate was selected, as it allowed for appropriate recovery of the fluorescence signal.

The pH of the medium plays a crucial role in modulating the properties of carbon dots, particularly when they are used as sensors. This is because the pH can directly impact the ionization of functional groups on the surface of the CDs, such as carboxyl, amine, or phenolic groups.^62^ The ionization of these groups influences the surface charge, stability, fluorescence emission, and the interactions of the CDs with target ions or molecules. Thus, proper pH control is crucial for optimizing the sensitivity and selectivity of the CDs, enhancing their efficiency in detecting specific analytes.^52^

To study the effect of pH, the CD dispersions were adjusted with appropriate amounts of 0.1 mol L^–1^ HCl or NaOH. Subsequently, Fe^3+^ and then glyphosate solutions were added to evaluate the influence of pH on the sensor performance during the two stages: fluorescence quenching by Fe^3+^ and fluorescence recovery upon glyphosate addition. Figure 8 illustrates the two stages of the study across the entire pH range investigated. In general, all CD samples exhibited significant decreases in fluorescence intensity in the presence of Fe^3+^ ions, along with fluorescence recovery that depends on both the specific system and the pH of the medium.

**Figure 8:**
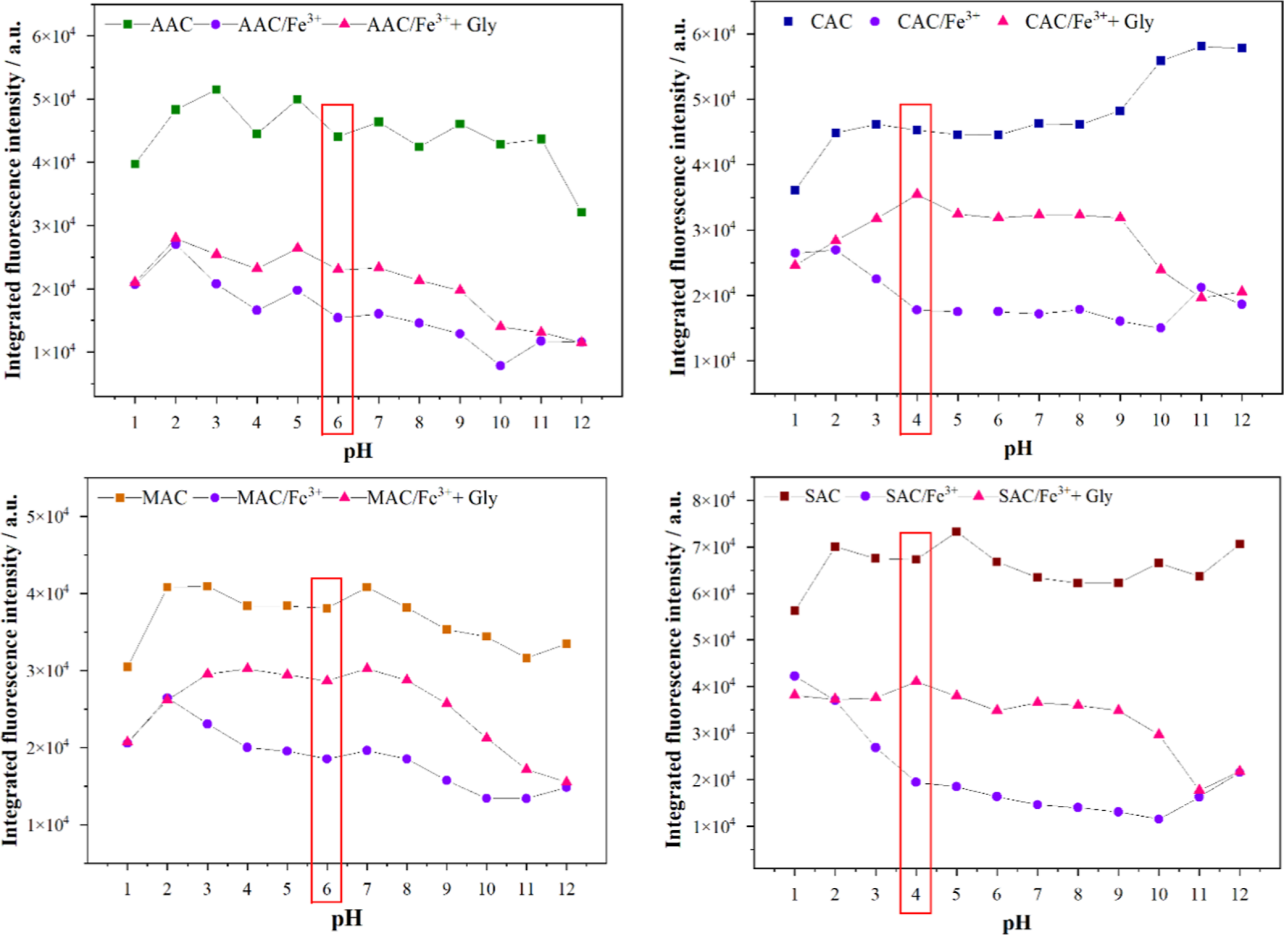
Effect of pH (1–12) on fluorescence intensity in CD, CD/Fe^3+^, and CD/Fe^3+^/glyphosate systems.

Fluorescence recovery of the CDs in the presence of glyphosate was observed under most conditions, except at extreme pH values (pH < 3 or >10), where PL restoration was negligible or absent. This behavior can be attributed to a combination of factors that directly affect the interactions among the CDs, Fe^3+^ ions, and glyphosate. In strongly acidic media, aggregation of the CDs may occur, hindering their interactions with Fe^3+^ and glyphosate. Moreover, the high concentration of H^+^ ions competes with Fe^3+^ for coordination sites. Additionally, under these conditions, glyphosate is predominantly protonated, which reduces its ability to coordinate with metal ions. On the other hand, in strongly alkaline media, Fe^3+^ undergoes hydrolysis, forming insoluble species such as Fe­(OH)_3_, which leads to precipitation and a consequent reduction in the concentration of free Fe^3+^ in solution. In the absence of available Fe^3+^, interaction with glyphosate becomes unfeasible, preventing fluorescence recovery. Although glyphosate predominantly exists in its trianionic form at high pH, with a high coordination potential, the lack of soluble Fe^3+^ limits the efficiency of this process.

More important, over a broad pH range (4–10), the CAC, MAC, and SAC samples exhibited considerable fluorescence recovery, whereas this effect was less pronounced in the AAC sample. These results highlight a significant advantage of these nanosensors, as their reliable performance across a broad pH range eliminates the need for precise pH control, thereby facilitating their application under varying sample conditions.

In addition, the optimal pH for glyphosate detection was determined by combining the highest fluorescence quenching efficiency by Fe^3+^ ions with the most effective fluorescence recovery in the presence of glyphosate, as shown in Figure 8. The differences observed in the results presented in Figure 8 can be attributed to slight variations in the pK_a_ values of the carboxylic groups on the surfaces of the different CDs, as the interaction is favored when these groups are deprotonated. In other words, the surface-bound carboxylic groups of the CDs may exhibit slightly different pK_a_ values depending on the local microenvironment, electronic effects, or hydrogen-bonding interactions, although they remain within the same order of magnitude. These variations affect the extent of interaction between the CDs and ferric ions and, consequently, can either facilitate or hinder the subsequent interaction of these ions with the herbicide. Thus, based on the results presented in Figure 8, the selected pH values for the glyphosate detection experiments were pH 6 for the AAC and MAC samples, and pH 4 for the CAC and SAC samples. The next section presents more detailed and quantitative results on glyphosate detection using the different prepared CDs under the established optimal conditions.

#### 3.2.3 Glyphosate Detection under Optimized Conditions

To quantitatively evaluate the restoration of fluorescence intensity, the CDs/Fe^3+^ system was exposed to increasing concentrations of glyphosate. For this analysis, the detection procedure followed the previously established optimized conditions. Initially, the sensor was prepared from a CD dispersion, with the pH adjusted to 4 for the CCA and SAC samples, and to 6 for the CAA and CMA samples. Subsequently, a Fe^3+^ solution was added, and the mixture was stirred using a magnetic stirrer for 3 min. Different volumes of glyphosate solution were then added, followed by a 10 min incubation period, after which the fluorescence intensity was measured. The specific concentrations and quantities used at each step are detailed in the Experimental section.

Figure 9 shows the effect of adding small amounts of glyphosate to the different CD/Fe^3+^ systems studied. The addition of the herbicide resulted in a gradual and linear increase in fluorescence intensity for all samples, as evidenced by the rise in *F*_1_/*F* values, where *F*_1_ and *F* correspond to the fluorescence intensities of the CDs/Fe^3+^ systems in the presence and absence of glyphosate, respectively. For each CD/Fe^3+^ system, slightly different concentration ranges were used due to their distinct behavior at low concentrations. The SAC/Fe^3+^ complex exhibited greater sensitivity to the presence of glyphosate, allowing the use of lower herbicide concentrations in this particular case (Figure 9). The LOD values for the samples were calculated using the 3δ/*S* formula, where δ represents the standard deviation and *S* is the slope of the curve. The resulting LODs were 1.57, 4.64, 11.16, and 0.59 μM for the AAC, CAC, MAC, and SAC samples, respectively. The LOD value obtained for the SAC sample, 0.59 μM (0.1 ppm), is adequate for detecting glyphosate at concentrations consistent with the maximum limits permitted in drinking water by various international regulations. In the United States, the established limit is 4.14 μM (0.7 ppm), whereas in Japan the maximum permissible concentration is 11.83 μM (2 ppm). In Brazil, the limit is set at 2.96 μM (0.5 ppm), whereas in China it is equivalent to the U.S. standard.^63^ Thus, the CD-based sensor provides sufficient sensitivity for detecting glyphosate at concentrations lower than those permitted by international standards.

**Figure 9:**
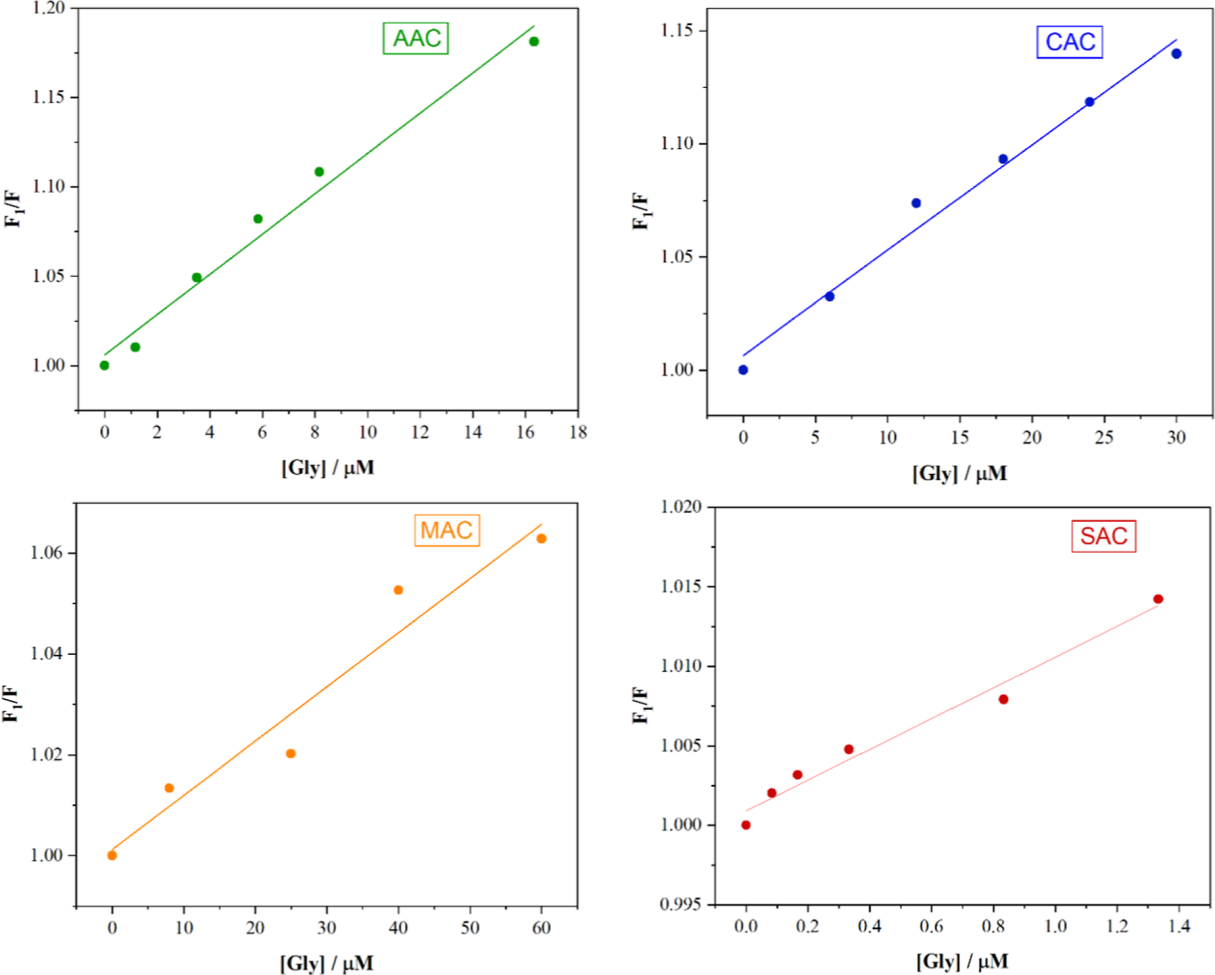
Effect of different glyphosate concentrations on the restoration of PL intensity in the CD/Fe^3+^ systems, evaluated by the *F*_2_/*F*_1_ ratio, where *F*_2_ and *F*_1_ represent the fluorescence intensity in the presence and absence of glyphosate, respectively.

Also, to investigate the photostability of the SAC sample, an experiment was conducted to monitor its PL intensity as a function of time, and the results are presented in Figure S11. The data indicate that the CD sample exhibits good stability in PL intensity, with no significant photobleaching observed during the investigated period. Furthermore, it is worth noting that, under practical conditions, the samples are stored in amber bottles to avoid any possible degradation caused by light exposure, and during sensor operation, they are exposed to radiation for a much shorter time than in the experiment shown in Figure S11. In addition, the minor fluctuations in PL intensity observed in Figure S11 (which are also characteristic of the instrument itself) are considerably smaller than the quenching induced by the CD/Fe^3+^ interaction or the fluorescence recovery observed in the presence of the herbicide. Therefore, it can be concluded that the SAC sample exhibits long-term photostability suitable for sensor applications.

In addition, it is important to note that, although traditional analytical methods for glyphosate detection, such as chromatography and mass spectrometry, provide very low detection limits (Table S2), these methods present certain limitations, including the need for highly specialized instruments, complex and time-consuming procedures, high costs, and skilled personnel. In contrast, fluorescence-based detection methods present several advantages, including simplicity, shorter response times, no requirement for sophisticated reagents or derivatization, and lower costs. Also, compared to other studies using CDs for glyphosate sensing, our system shows competitive performance (Table S2). While some reports have presented lower LODs (e.g., Hou et al.,^60^ 0.0087 ppm, and Li et al.,^64^ 0.0021 ppm) and others similar or higher values (e.g., Yuan et al.,^65^ 0.101 ppm), the LOD obtained in this work remains well below relevant regulatory limits. The SAC sample also exhibits a relatively low working range (0.0135–0.237 ppm), which is advantageous as it enables accurate quantification at lower concentrations. Also, unlike most studies that use Cu^2+^ as the quencher, we employed Fe^3+^, which provided high selectivity, and studied the interaction mechanism (static quenching) in detail. The system further demonstrated tolerance to variations in ionic strength, effective performance across pH 4–10, and rapid response (3 min for quenching; 10 min for recovery). Overall, the sensitivity, wide working range, robustness, and fast response indicate that the proposed system is practical for environmental monitoring. Thus, the method has the potential to be employed in rapid, low-cost, and environmentally friendly tests, making it a promising alternative for monitoring the herbicide in aquatic environments.

Based on the results obtained for the SAC sample, which exhibited higher selectivity and sensitivity toward Fe^3+^ ions, lower susceptibility to ionic strength, and the lowest detection limit for the herbicide, this sample was chosen for application of the method to a real tap water sample.

#### 3.2.4 Applications in Real Samples

To evaluate the applicability of the sensor in real samples, a 20 ppm of SAC dispersion was prepared in a 3.0 mL volume, with the pH adjusted to 4. Then, 30 μL of a 0.01 mol·L^–1^ Fe^3+^ solution was added, and the mixture was stirred for 3 min to ensure thorough homogenization and interaction between the sensor components. Two types of real samples were collected: tap water obtained directly from the laboratory and water from a local urban stream in Belo Horizonte, Brazil. To ensure analytical quality and minimize potential interferences, the samples were prefiltered through a 0.22 μm membrane to remove suspended solids. Spiking experiments were then conducted using these real samples. The filtered samples were spiked with different volumes of a 0.01 mol·L^–1^ glyphosate standard solution, as summarized in Table 2, and then incubated for 10 min. Quantification of the analyte was carried out using the calibration curves (Figure S12), within the concentration range of 3.3–66.7 μM for both types of water samples. All measurements were performed in triplicate (*n* = 3).

**Table 2:** Glyphosate Determination in Spiked Real Water Samples (*n* = 3)

sample	amount added/μM	amount found/μM	recovery/%	RSD/%
**tap water**	0	0	-	0.92
	6.7	6.5	98.3	0.74
	16.7	17.0	102.4	0.43
	33.3	32.4	97.2	0.42
	66.7	67.0	100.4	0.95
**urban streamwater**	0	0	-	0.16
	16.7	15.9	95.5	0.90
	33.3	34.6	103.6	1.11
	50.0	48.2	96.4	1.50
	83.3	81.7	98.0	0.89

As summarized in Table 2, the recoveries for spiked tap water and river water ranged from 97.2 to 102.4% and 95.5 to 103.6%, respectively, demonstrating good precision and accuracy of the method. The highest relative standard deviation (RSD) observed was 1.50%. These results confirm that the proposed fluorescent sensor is a reliable tool for glyphosate detection in real water samples, indicating adequate sensitivity for applications in aqueous environments.

## 4 Conclusion

In this work, we developed an “off-on” sensor for the detection of the herbicide glyphosate, based on Fe^3+^-mediated fluorescence quenching and recovery. To this end, we initially prepared and compared the properties of four different CDs obtained via hydrothermal carbonization.

All obtained CDs exhibited an *n* → π* transition arising from oxygen- and nitrogen-containing surface groups, whereas the AAC sample also displayed a π → π* transition characteristic of C=C conjugation in aromatic systems. The nanoparticles exhibited strong emission in the blue-green region of the spectrum, with the samples CAC and SAC showing the highest QY value of 21.6%.

The SAC sample combined the most favorable properties for use as a glyphosate sensor, including improved photoluminescence stability under varying ionic strength conditions, along with enhanced selectivity and sensitivity toward ferric ions (LOD = 0.37 μM). Furthermore, the quenching mechanism of this sample toward ferric ions was determined to be static, suggesting the formation of a nonfluorescent ground-state complex.

A detailed study was carried out to optimize the experimental conditions for glyphosate detection. The optimal pH was 4 for the CAC and SAC samples, and 6 for the AAC and MAC samples, while the incubation times for Fe^3+^ (quenching) and glyphosate (fluorescence recovery) were 3 and 10 min, respectively. Under optimized conditions, the SAC/Fe^3+^ system achieved a glyphosate LOD of 0.59 μM and provided accurate results for spiked aqueous samples from tap and an urban stream, with recoveries ranging from 97.2 to 102.4% and 95.5 to 103.6%, respectively, and RSD ≤ 1.50%. These results indicate that the developed approach is suitable for simple aqueous matrices without the need for extensive sample pretreatment.

Taken together, the results indicate that tuning CD precursor chemistry and controlling assay conditions yields reproducible fluorescence-based sensors for glyphosate, featuring rapid response and detection limits below international regulatory thresholds, providing an accessible and environmentally friendly option for monitoring the herbicide in aquatic environments.

## Supplementary Material



## Notes

Additional PL emission spectra; QY determination; particle size distribution; Raman spectra; X-ray diffraction patterns; elemental analysis; effect of salt concentration; PL intensity interference caused by different metal ions; additional fluorescence emission profiles; Stern–Volmer plots; fluorescence lifetime decay curves; PL intensity as a function of time; analytical performance of reported glyphosate detection methods; calibration curves of the integrated fluorescence intensity; and additional references are provided in the Supporting Information section (PDF)
