# Supplementary material for: Carbon Dots-Based Fluorescent Sensor for Glyphosate Detection Via Fe3+-Mediated Fluorescence Quenching and Recovery
Source: ACS Omega. 2025 Dec 2;10(49):60844–58. doi: 10.1021/acsomega.5c09330 (PMC12713492; doi:10.1021/acsomega.5c09330)
Supplement: Supplementary file 1 [file ao5c09330_si_001.pdf]

Carbon dots-based fluorescent sensor for glyphosate detection via Fe^{3+}-mediated fluorescence quenching and recovery

*Gabriela Fernandes Barreto, Ricardo Mathias Orlando, Fabiano Vargas Pereira**

Department of Chemistry – Universidade Federal de Minas Gerais. Av. Antônio Carlos, 6627 - Pampulha - Belo Horizonte - MG, Brazil. CEP 31270-901

* *fabianovp@ufmg.br*

Supplementary Material

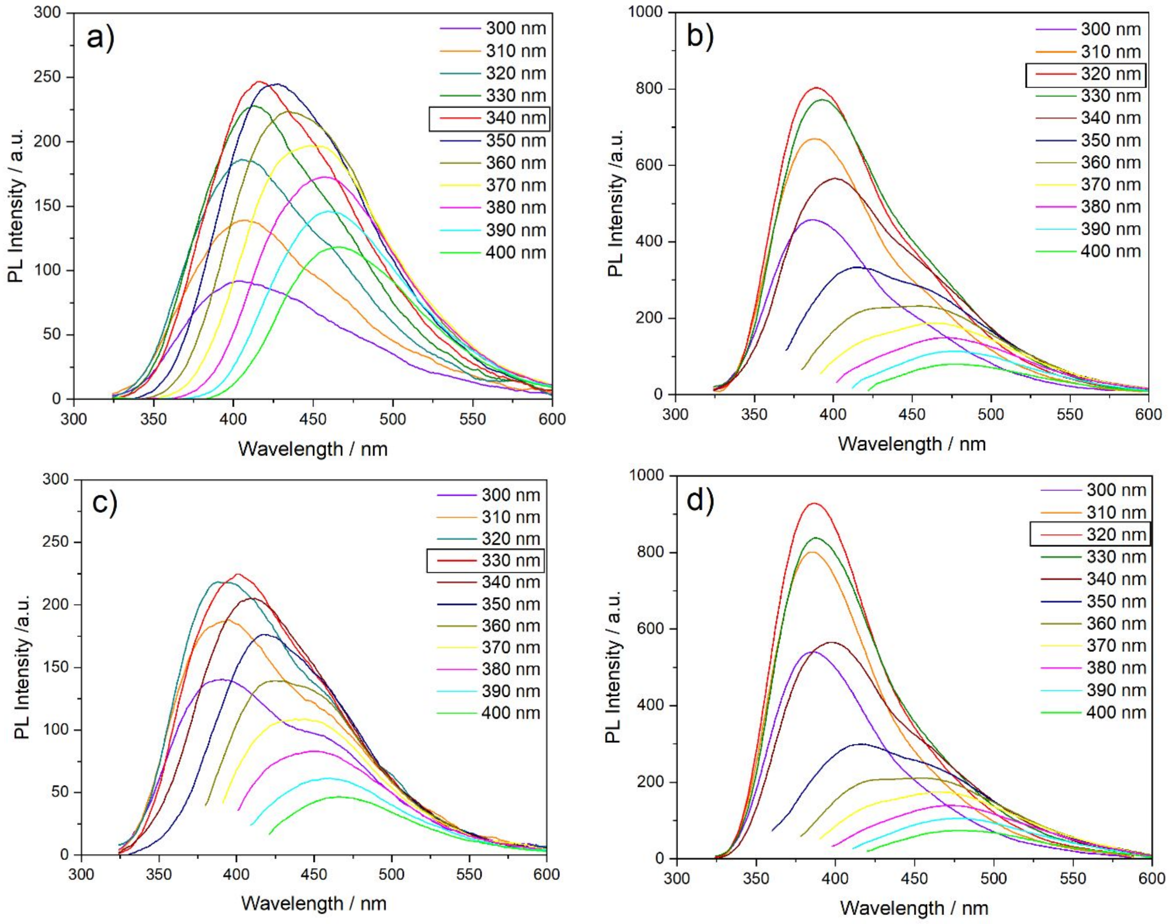

Figure S1: Non-normalized PL emission spectra of the samples: (a) AAC, (b) CAC, (c) MAC, and (d) SAC. Boxed values indicate the excitation wavelengths corresponding to maximum emission for each sample.

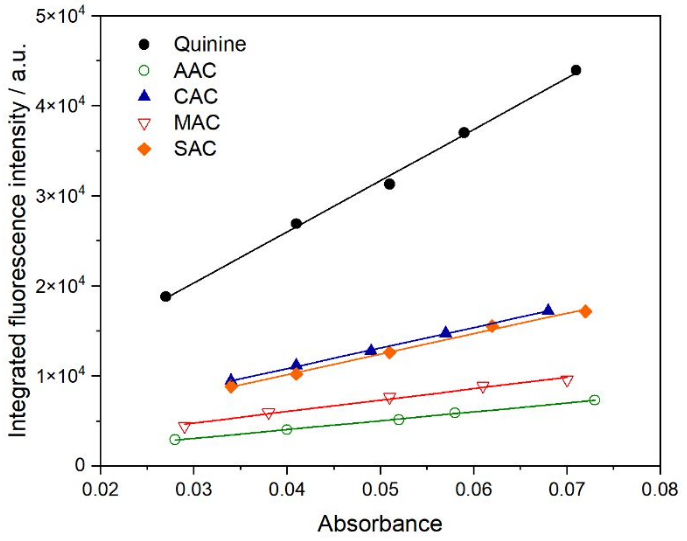

Figure S2: Fluorescence intensity and absorbance values of quinine sulfate (standard) and the CDs for QY determination.

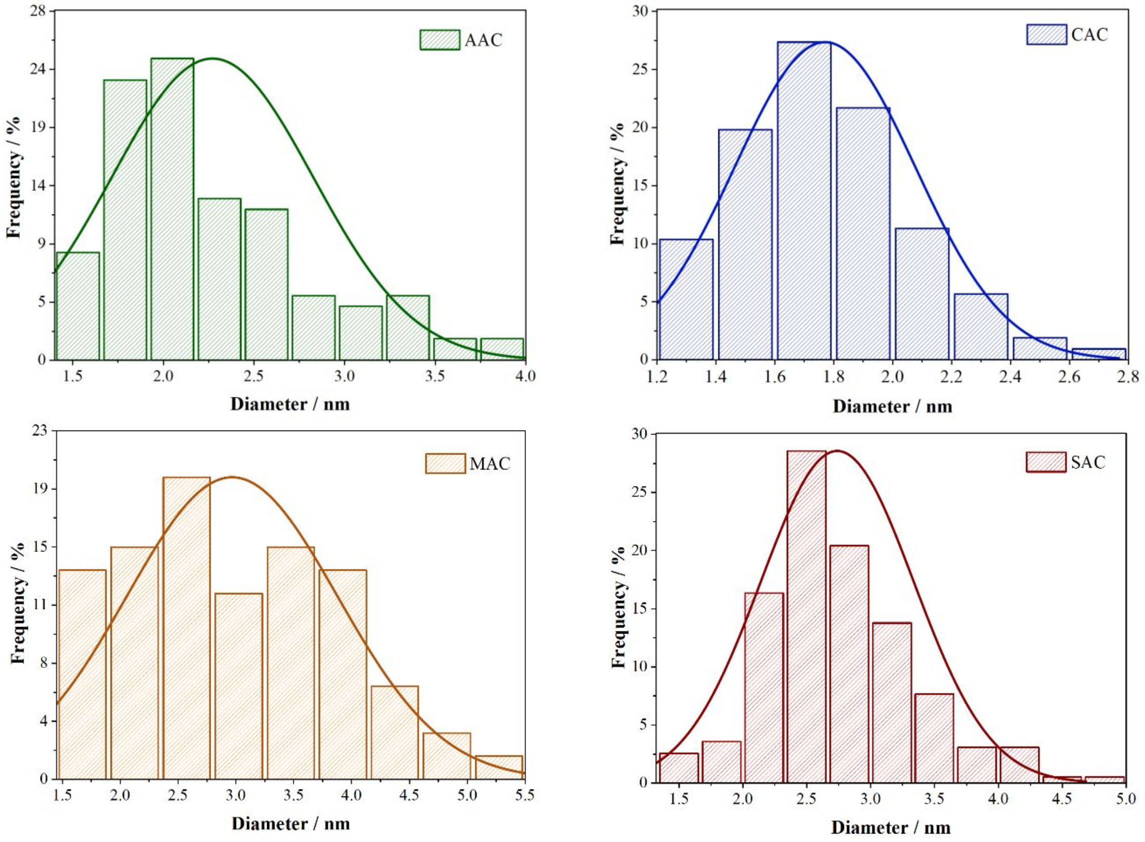

Figure S3: Particle size distribution histograms of the CD samples.

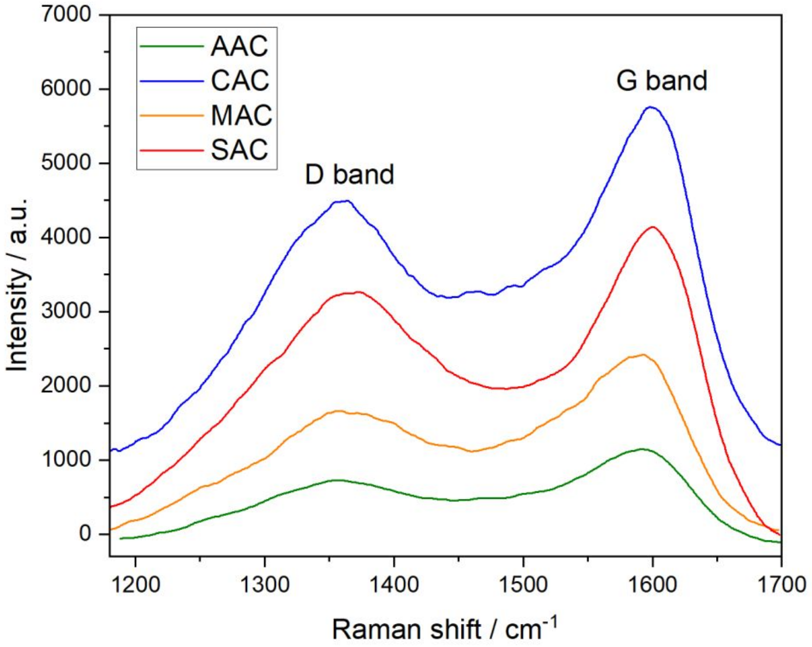

Figure S4: Raman spectra of the CDs samples.

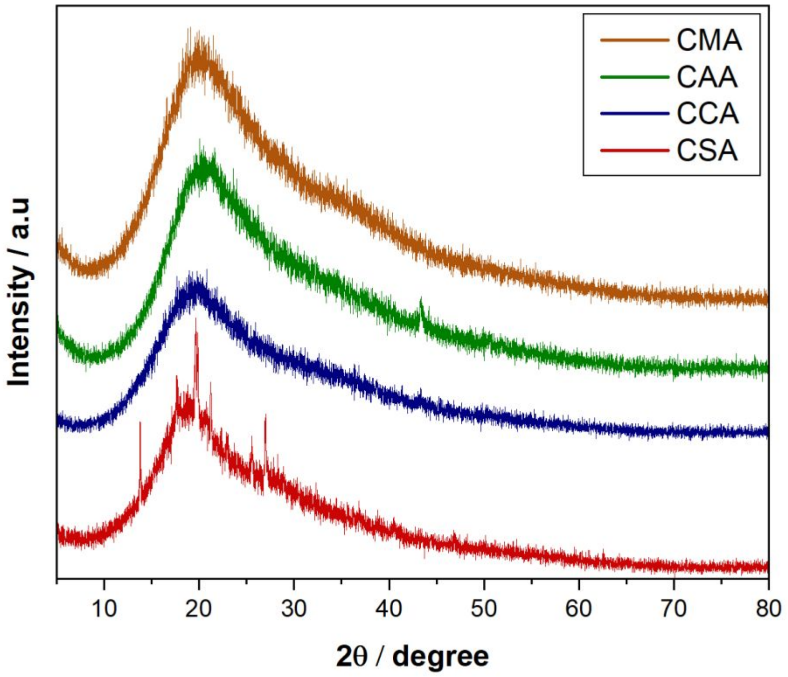

Figure S5: X-ray diffraction of the CD samples

Table S1: Elemental analysis results of the CD samples.

**Samples**	**C / %**	**H / %**	**N / %**	**O / %**
**AAC**	55.22 ± 0.03	5.19 ± 0.01	9.72 ± 0.01	29.88 ± 0.04
**CAC**	52.92 ± 0.67	5.13 ± 0.02	10.38 ± 0.16	31.59 ± 0.86
**MAC**	51.44 ± 0.15	4.58 ± 0.01	10.81 ± 0.02	33.18 ± 0.18
**SAC**	53.09 ± 0.08	5.08 ± 0.01	11.78 ± 0.02	30.06 ± 0.11

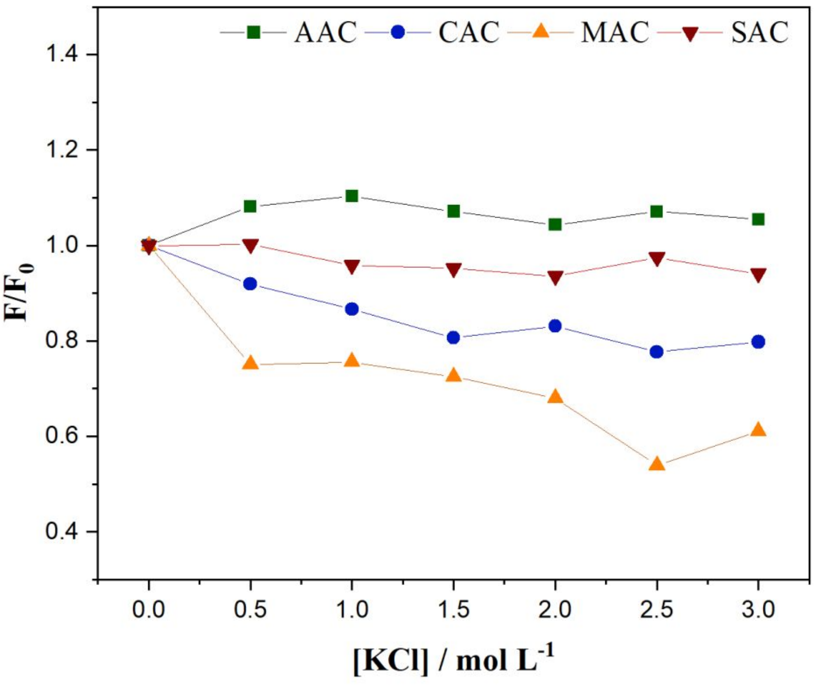

Figure S6: Effect of salt concentration on the fluorescence intensity of the different CDs.

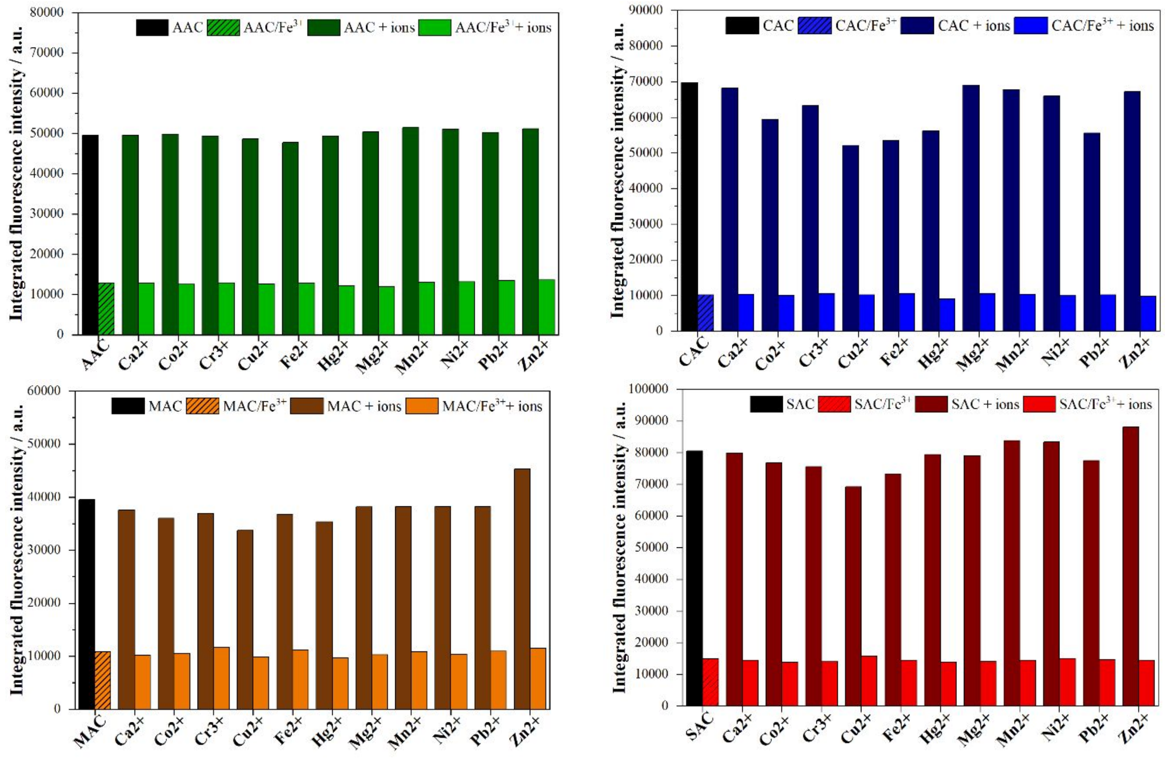

Figure S7: Evaluation of fluorescence intensity interference caused by the addition of different metal ions to the CDs/Fe^{3+} system for the samples.

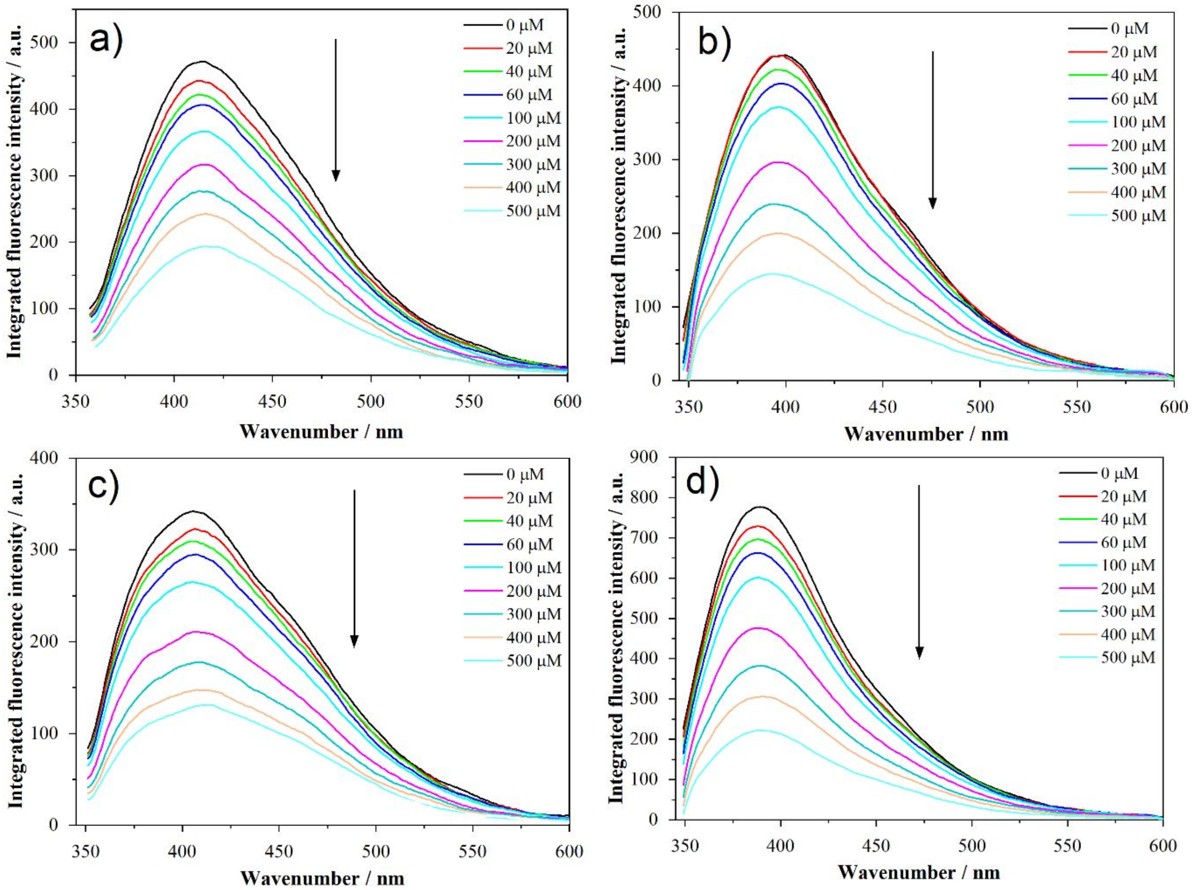

Figure S8: Fluorescence emission profiles of the CD samples in the higher Fe^{3+} concentration range (200-500 μM), excited at the wavelength corresponding to the maximum emission for each sample: a) AAC, b) CAC, c) MAC, and d) SAC.

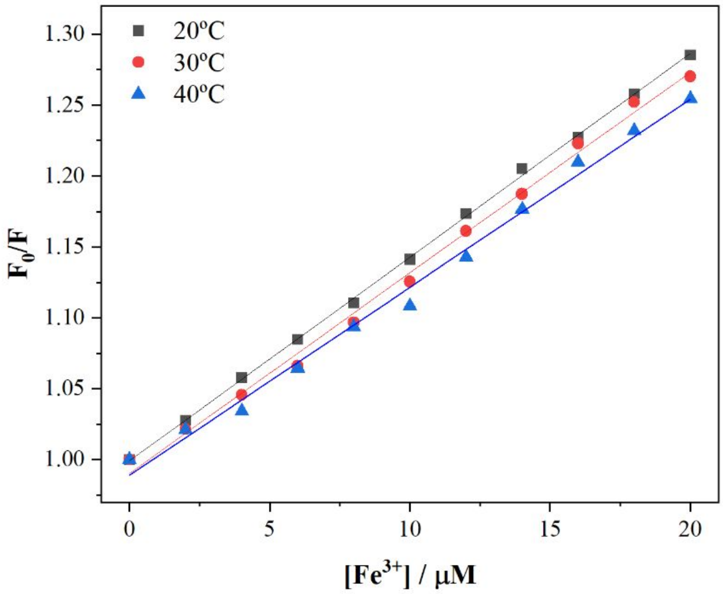

Figure S9: Stern-Volmer plot of the SAC sample in the presence of varying Fe^{3+} concentrations at different temperatures. F_0 and F represent the fluorescence intensities in the absence and presence of Fe^{3+}, respectively.

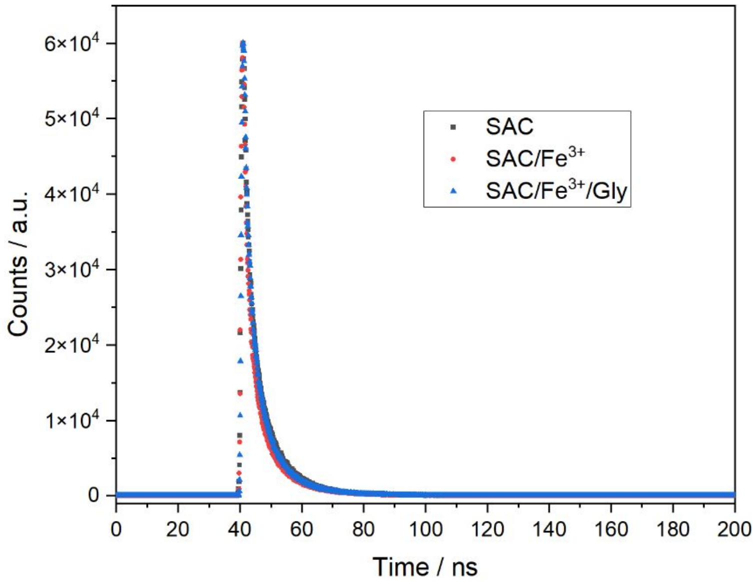

Figure S10: Fluorescence lifetime decay curves of SAC, SAC/Fe^{3+}, and SAC/Fe^{3+}/glyphosate samples.

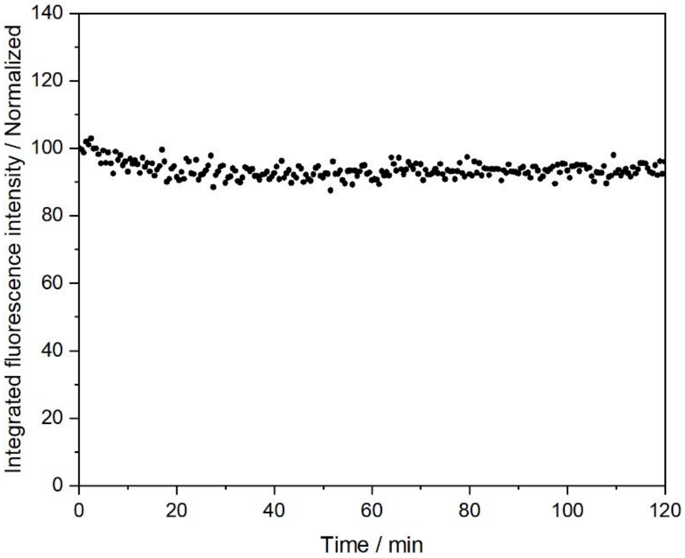

Figure S11: PL intensity (excitation at 320 nm) as a function of time for the SAC sample.

Table S2: Analytical performance of reported glyphosate detection methods, including carbon dot-based sensors.

Method	LOD / ppm	Linear range / ppm	Reference
LC-MS/MS	0.002	0.002-0.5	RS1*
HPLC-ESI-MS/MS	$5.0x10^{-5}$	10^{-4}-0.1	RS2*
LC-MS/MS	$8.0x10^{-5}$	10^{-3}-0.2	RS3*
UHPLC-MS/MS	$2.6x10^{-5}$	10^{-4}-0.4	RS4*
Fluorescence/CD	0.0087	0.1-16	60
Fluorescence/CD	0.0021	0.1-10	64
Fluorescence/CD	0.101	0.34-4.0	65
Fluorescence/CD	0.100	0.0135-0.237	This work

*The references labeled RS1-RS4 correspond to studies listed in the Supplementary Material; all other references are provided in the main manuscript.

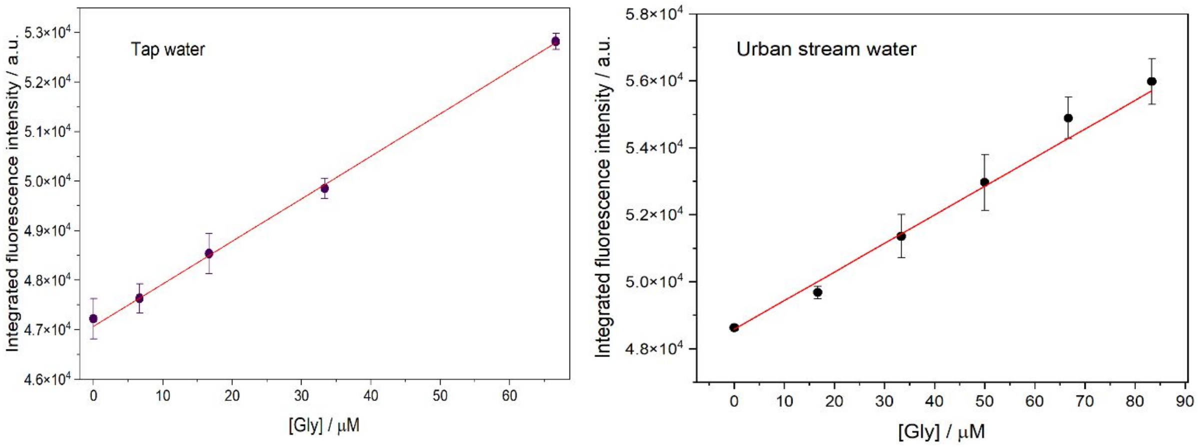

Figure S12: Calibration curves of the integrated fluorescence intensity as a function of glyphosate concentration for tap water and urban stream samples.

References:

(RS1) Fritz-Wallace, W.; Engelmann, B.; Krause, J.L.; Schäpe, S.S.; Pöppe, J.; Herberth, G.; Rösler, U. et al. Quantification of glyphosate and aminomethylphosphonic acid from microbiome reactor fluids. *Rapid Communications in Mass Spectrometry* **2020**, 34, e8668. DOI: 10.1002/rcm.8668.

(RS2) Guo, H.; Riter, L.S.; Wujcik, C.E.; Armstrong, D.W. Direct and sensitive determination of glyphosate and aminomethylphosphonic acid in environmental water samples by high performance liquid chromatography coupled to electrospray tandem mass spectrometry. *Journal of Chromatography A* **2016**, 1443, 93-100. DOI: 10.1016/j.chroma.2016.03.020

(RS3) Ulrich, J.C.; Ferguson, P.L. Development of a sensitive direct injection LC-MS/MS method for the detection of glyphosate and aminomethylphosphonic acid (AMPA) in hard Waters. *Analytical and Bioanalytical Chemistry* **2021**, 413(14), 3763–3774. DOI: 10.1007/s00216-021-03324-5

(RS4) Brown, A.K.; Farenhorst, A. Quantitation of glyphosate, glufosinate, and AMPA in drinking water and surface waters using direct injection and charged-surface ultra-high performance liquid chromatography-tandem mass spectrometry **2024**, 349, 140924. DOI: 10.1016/j.chemosphere.2023.140924
